# Neuroblastoma cell lines display heterogeneity in differentiation responses

**DOI:** 10.12688/wellcomeopenres.23249.2

**Published:** 2025-09-16

**Authors:** Kirsty M. Ferguson, Sarah L. Gillen, Fiona M. Y. Abou Grealy, Aditi Vedi, Anna Philpott

**Affiliations:** 1 Jeffrey Cheah Biomedical Centre, Cambridge Stem Cell Institute, Cambridge, England, CB2 0AW, UK; 2 Cambridge University Hospitals NHS Foundation Trust, Cambridge, England, UK; 3 University of Cambridge Department of Paediatrics, Cambridge, England, CB2 0XZ, UK; 4 University of Cambridge Department of Oncology, Cambridge, England, CB2 0XZ, UK

**Keywords:** neuroblastoma, differentiation, palbociclib, retinoic acid, neural crest, extracellular matrix

## Abstract

**Background:**

Neuroblastoma is the most common extra-cranial childhood solid tumour, arising during development from stalled neural crest-derived precursor cells. In a subset of children younger than 18 months of age, neuroblastoma can undergo spontaneous regression driven by differentiation, leading to great interest in developing differentiation therapies to re-direct neuroblastoma cells down their correct developmental pathway. Recently, we have shown that combinatorial treatment with the CDK4/6 inhibitor palbociclib and differentiation-inducing agent retinoic acid inhibits proliferation and drives neuronal differentiation of adrenergic-type neuroblastoma cell lines.

**Methods:**

Here, we explore the differentiation potential of neuroblastoma cell lines *in vitro* in response to palbociclib and retinoic acid treatment using microscopy, transcriptomic and qRT-PCR analyses.

**Results:**

We present evidence suggesting that neuroblastoma cells can give rise to mixtures of neural crest-derived adrenal gland cell types, and that differentiation responses correspond to changes in patterns of extracellular matrix substrate expression.

**Conclusions:**

This study builds a case to further investigate and consider heterogeneity in neuroblastoma cell differentiation and extracellular matrix changes during these cell fate decisions.

## Plain language summary

Neuroblastoma is a childhood cancer that originates from developing cells of the nervous system outside the brain; these cells do not develop according to plan and take the wrong path. We have previously found that two drugs, namely palbociclib and retinoic acid, can together push neuroblastoma cells to specialise into neurons. However, neuroblastomas are known to be made up of diverse cell types and their relationship to the normal developing adrenal gland, where tumours are thought to originate, has been extensively studied recently.

In this research article, we wanted to see how treatment with palbociclib and retinoic acid affects the “development pathways” taken by neuroblastoma cells. We find that neuroblastoma cells from different patients follow different paths in response to this drug treatment. Not only that, we present evidence that neuroblastoma cells from the same patient can take multiple paths to form different cell types. In addition, these different responses are linked to changes in the extracellular matrix, a network of molecules that provides structural support to cells in the body.

Together these data suggest the scientific community should further investigate the potential of neuroblastoma cells to specialise down different paths, as well as consider changes outside the cell in these pathway decisions.

## Introduction

Neuroblastoma is the most common extracranial solid tumour in infants, arising from stalled neural crest cells, with the majority centred in the adrenal glands and sympathetic ganglia ^1^. Neural crest-derived sympathoadrenal precursor cells differentiate into cell types including sympathetic neurons and adrenal chromaffin cells during normal development. Neuroblastoma originates when sympathetic precursor cells do not undergo correct differentiation remaining in an immature state that drives tumour growth ^2, 3^. A subset of tumours (International Neuroblastoma Risk Group (INRG) stage MS) in infants under 18 months of age can undergo spontaneous remission linked to tumour cell differentiation ^4– 6^. Neuroblastoma therefore presents a unique opportunity whereby differentiation therapies that reactivate normal developmental processes provide a promising therapeutic approach.

Recently, we have shown that cell cycle lengthening with the CDK4/6 inhibitor palbociclib promotes differentiation in neuroblastoma cell lines. Furthermore, combinatorial treatment with palbociclib and the differentiation-inducing agent retinoic acid potentiates this effect by inhibiting proliferation and driving neuronal differentiation of adrenergic-type (ADRN) neuroblastoma cell lines ^7^. Differentiation is considered to be along the sympathoadrenal lineage as cells are locked in an immature progenitor state along this pathway, marked by features such as neurite extension. However, another cell type, flat and substrate adherent, was previously reported in neuroblastoma cell lines treated with retinoic acid and dbc-AMP ^8^. Neuroblastoma cells have been found to express distinct neural crest lineages ^9^ and bidirectional differentiation of neuroblastoma cell lines in response to retinoic acid and staurosporine has also been reported ^10^. Furthermore, neural crest cells have also been shown to retain their ability for multipotential differentiation even after lineage-restricted stages ^11^. Neuroblastoma cultures are indeed heterogeneous, and each cell line may consist of cells stalled at different stages of developmental maturation along the neural crest lineage. In the clinical setting, this translates to significant intra-tumoural heterogeneity within the same patient. This phenotypic heterogeneity (N/S/I-type) and the underlying transcriptional core regulatory circuitries (adrenergic (ADRN) and mesenchymal (MES)) has been extensively reviewed in vitro, with evidence found of transcriptional plasticity and trans-differentiation between cell types ^12– 16^. In keeping with the developmental role of the extracellular matrix (ECM) in neural crest differentiation and migration ^17^, Tsokos *et al.* ^8^ reported that induced differentiation responses of different neuroblastoma cell lines correspond to specific changes to ECM substrate expression proportional to the normal tissue cell types that emerge.

Here, we explore the developmental potential of ADRN neuroblastoma cell lines *in vitro* in response to palbociclib and retinoic acid treatment. We present evidence suggesting that neuroblastoma cells can give rise to mixtures of neural crest-derived adrenal gland cell types, and that differentiation responses correspond to changes in patterns of ECM substrate expression. These data open up our view of neuroblastoma differentiation, beyond neuronal cell fates, and build a case for investigating ECM changes related to neuroblastoma differentiation.

## Methods

### Bioinformatics analyses

Genesets were derived from the clusters assigned in 7 based on changes in bulk RNA-seq expression of SK-N-BE(2)C cells with 5 days PB and RA treatment. For the genes in these lists that are detectable in the single cell dataset from 1, Seurat AddModuleScore was used to see how the enrichment of the average expression of these gene sets varies across the dataset and is displayed on the UMAPs generated with Seurat ^18^.

### Cell culture

Neuroblastoma cell lines SK-N-BE(2)C, IMR-32 and SH-SY5Y were cultured in DMEM-F12 with L-glutamine (Sigma, D8437) supplemented with 10% FBS (PAN-Biotech, P40-37500) and 1% Penicillin-Streptomycin (Sigma, P0781), with media refreshed every 2-3 days. Cells were confirmed to be *Mycoplasma* negative and tested at a minimum of every 3 months. Palbociclib (PD-0332991 HCl, SelleckChem, S1116) was dissolved in DMSO (Santa Cruz, sc-358801) and used at a final concentration of 1 μM. All-trans retinoic acid (Sigma, R2625) was dissolved in DMSO and used at a final concentration of 10 μM.

### Quantitative RT-PCR (qRT-PCR)

Cells were lysed using RLT buffer and RNA extracted using the RNeasy Mini kit (Qiagen, 74104). RNA concentration was determined using a NanoDrop™ Spectrophotometer. Reverse transcription was performed using the QuantiTect Reverse Transcriptase Kit (Qiagen, 205311). Quantitative RT-PCR (qRT-PCR) was performed using gene-specific oligonucleotide primers (Table S1, Extended data) and SYBR™ Green Master Mix (Applied Biosystems, A25742) on an Applied Biosystems StepOne™ Real-Time PCR system. Replicate Ct values were averaged and normalised to the housekeeping gene, *TBP*. Data are presented as Fold change (RQ), where this value equals one for the calibrator sample. Data presented as Mean +/- 95% CI, with error bars calculated from ddCt values prior to transformation.

### Statistical analysis

qRT-PCR statistical analyses were performed using GraphPad Prism software (version 10.2.2 for macOS, https://www.graphpad.com), as noted in the figure legends (*, *P* ≤ 0.05; **, *P* ≤ 0.01, ***, *P* ≤ 0.001; and ****, *P* ≤ 0.0001) from three independent experiments. R is a free software alternative. GraphPad Prism Viewer is free and allows visualisation of data, analyses and graphs. Biological replicates were considered as different passage numbers of the same cell line plated in independent experiments. Mean and 95% CIs, and n numbers, are shown in the figure legends. For qRT-PCR, statistics were performed on ddCt values prior to transformation.

## Results

### Transcriptomic analyses suggest SK-N-BE(2)C neuroblastoma cells have potential to enter different human adrenal developmental lineages

**Figure 1.**
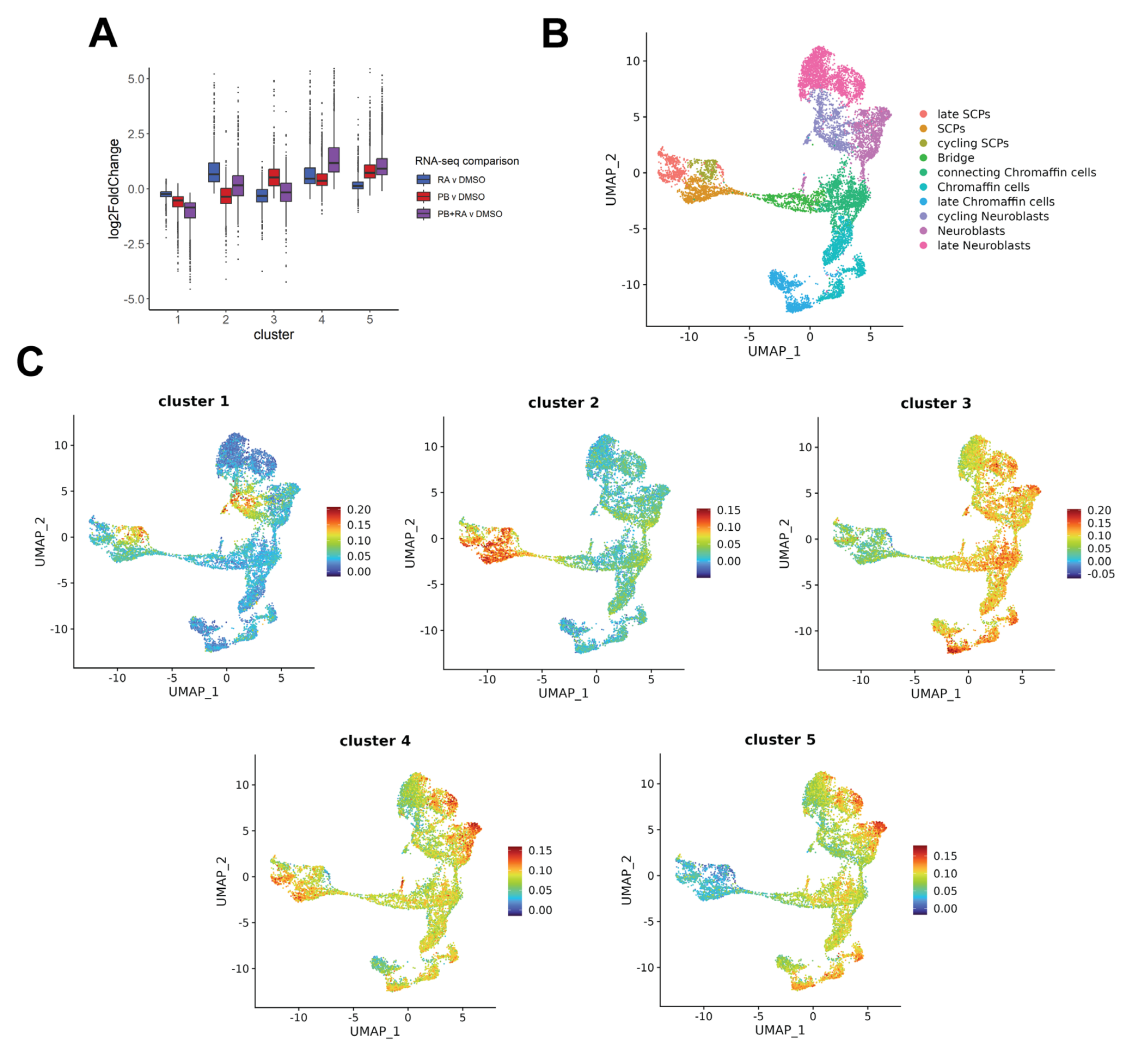
Transcriptomic analyses suggest SK-N-BE(2)C neuroblastoma cells have potential to enter different human adrenal developmental lineages. **A**) Boxplot displaying the log2-fold change in expression of gene clusters 1 to 5 in PB, RA or PB+RA treated SK-N-BE(2)C cells (5 days) compared to DMSO-treated control (RNA-seq data in Figure 6 of 7). Cluster 1: Genes associated with cell-cycle progression, Cluster 2: Genes associated with cell-cell junctions and extracellular matrix, Cluster 3: Genes associated with post-synaptic membrane components, Cluster 4: Genes associated with neuronal cell body and extracellular matrix, Cluster 5: Genes associated with synaptic vesicles, synaptic membranes and neuronal development. **B**) UMAP plot of adrenal medullary cells, adapted from Figure 1 of 1. Single cell populations are coloured according to their cell-type. **C**) UMAPs generated with Seurat displaying module scores for gene sets in each of the clusters (1 to 5, (A)) from 7 within the human foetal adrenal medulla single-cell dataset in 1.

Previously, we showed that combinatorial treatment with the CDK4/6 inhibitor palbociclib (PB) and differentiation-inducing agent retinoic acid (RA) inhibits proliferation and drives neuronal differentiation of ADRN neuroblastoma cell lines, to a greater extent than either agent alone ^7^. To characterise the effect of PB or PB+RA on neuroblastoma cells, RNA-seq analyses were previously performed on SK-N-BE(2)C cells treated with DMSO vehicle control, PB, RA or PB+RA for five days (see Figure 6 in 7). Following k-means clustering, five distinct gene clusters were identified with different responses to PB, RA or PB+RA treatment (Table S2, Extended data). Cluster 1, associated with cellular components involved in cell-cycle progression, encompassed genes downregulated by PB, RA or PB+RA treatment, whereas clusters 2, 3, 4 encompassed genes upregulated only in response to RA, PB or PB+RA, respectively ( Figure 1A). These clusters were associated with cell-cell junctions and the extracellular matrix (cluster 2), postsynaptic membrane components (cluster 3) and neuronal cell body and extracellular matrix cellular components (cluster 4). Cluster 5 marked a subset of genes upregulated by PB and further upregulated by PB+RA ( Figure 1A), associated with synaptic vesicles, synaptic membranes, and other components associated with neuronal development. The cell types of the developing adrenal medulla, thought to contain the cell of origin of neuroblastoma, and their lineage trajectories have recently been characterised by single-cell RNA-seq and compared to neuroblastoma transcriptomes ^1^. The cell type composition of SK-N-BE(2)C matched the adrenal medulla, and while treatment with all-trans retinoic acid (RA) reduced the proportion of cycling neuroblasts, most cells retained progenitor status ^1^. We wondered how combined PB+RA treatment might affect these developmental trajectories. Here, we investigated the expression of the genes in each of these clusters within a published human single-cell RNA-seq dataset for human adrenal medulla development ( Figure 1B) ^1^. The cluster 1 gene set, downregulated in all treatment conditions, is enriched in cycling neuroblast and cycling Schwann cell precursor (SCP) populations, in fitting with an expected decrease in cycling cell populations upon differentiation induction ( Figure 1C). While the gene set upregulated by RA treatment alone (cluster 2) is enriched in SCP cells, genes upregulated by PB alone (cluster 3) are enriched in connecting chromaffin cells, neuroblasts and (late) chromaffin cells ( Figure 1C). Interestingly, the PB+RA treatment gene signature (cluster 4) is enriched in late neuroblast and late SCP gene cells, while the PB or PB+RA treatment regulated gene set (cluster 5) is enriched in (late) neuroblast and (late) chromaffin cells ( Figure 1C). While further validation is required as detailed in the discussion, this suggests that SK-N-BE(2)C neuroblastoma cells may have the potential to enter different human adrenal medullary developmental lineages in response to PB+RA-induced differentiation, in addition to neuronal differentiation.

### Adrenergic neuroblastoma cells display heterogeneous differentiation responses to PB+RA

Given our RNA-seq analyses suggest SK-N-BE(2)C cells were able to enter non-neuronal lineages upon PB+RA treatment for 5 days, we next explored treating the ADRN neuroblastoma cell lines SK-N-BE(2)C, IMR-32 and SH-SY5Y with PB+RA for an extended period of 10 days. These cell lines, also used in our previous publication ^7^, represent ADRN-type cells with different genetic backgrounds: both SK-N-BE(2)C and IMR-32 are MYCN amplified and ALK wildtype, while SH-SY5Y is MYCN wildtype and ALK mutant (F1174L). SK-N-BE(2)C was derived from a bone marrow biopsy of a relapsed neuroblastoma following chemotherapy ^19^, while SH-SY5Y is a relapsed subline of SK-N-SH established from a metastatic bone tumour of a neuroblastoma patient ^20^ and IMR-32 was derived from a neuroblastoma abdominal mass at diagnosis ^14^. Whilst all are ADRN-type cells, SK-N-BE(2)C are phenotypically characterised as intermediate ‘I’ type, expressing both features of neuroblastic (N) and substrate-adherent (S)-type cells ^21^, while SH-SY5Y and IMR-32 are neuroblastic ‘N’ type ^14^.

**Figure 2.**
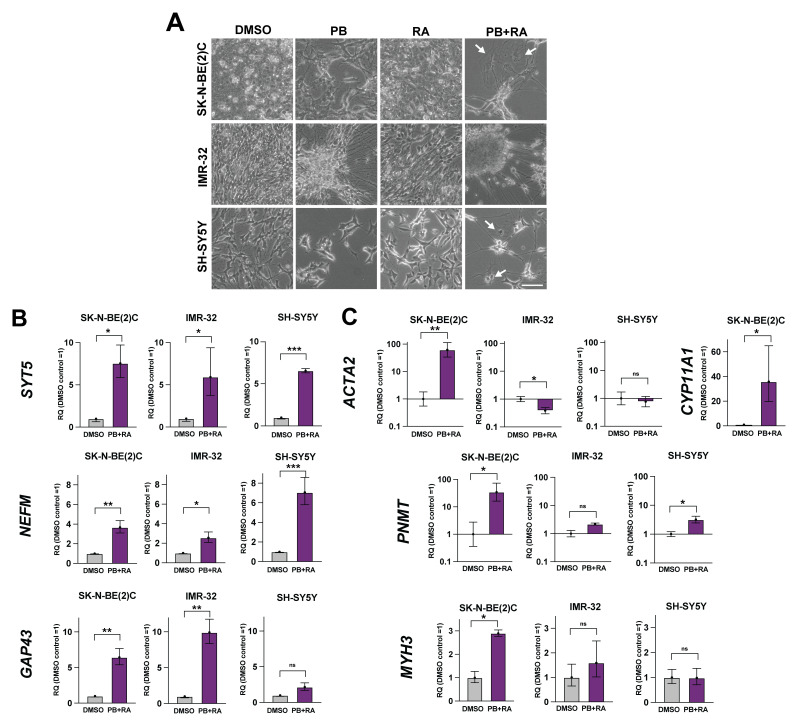
Adrenergic neuroblastoma cells display heterogeneous differentiation responses to PB+RA. **A**) Phase-contrast images of SK-N-BE(2)C, IMR-32 or SH-SY5Y cells treated with DMSO, palbociclib 1 μM (PB), all-trans retinoic acid (10 μM, RA) or PB+RA for 10 days. Representative of n = 3 biological replicates. Scale bar: 100 μm. Videos depicting the untreated cell morphology at starting confluence can be viewed in Videos S1-6 in 7 for reference. **B**) qRT-PCR analysis of *SYT5, NEFM* and *GAP43,* and **C**) *ACTA2, CYP11A1, PNMT* and *MYH3* expression levels in SK-N-BE(2)C, IMR-32 or SH-SY5Y cells treated with DMSO or PB+RA (PB 1 μM + RA 10 μM) for 10 days. n = 3 biological replicates. Biological replicates were considered as different passage numbers of the same cell line plated in independent experiments. Note: No detectable *CYP11A1* expression in IMR-32 or SH-SY5Y cells. Mean RQ ± 95% CI. *p ≤ 0.05; **p ≤ 0.01, ***p ≤ 0.001; and ****p ≤ 0.0001. Two-tailed unpaired t-test.

Strikingly, each cell line displayed a different differentiation response ( Figure 2A). While IMR-32 displayed only features of neuronal differentiation, with the formation of large clusters of differentiating cells connected by extensive networks of neurites, SK-N-BE(2)C and SH-SY5Y appeared to enter additional cell fates with flattened morphology ( Figure 2A). Select qRT-PCR analyses were used to further explore the emerging cell fates, based on markers of adrenal gland development in 1. All three lines showed an upregulation in expression of the sympathetic neuroblast markers *NEFM* and *SYT5* by qRT-PCR following PB+RA treatment compared to their respective DMSO-treated controls ( Figure 2B). Expression of *GAP43*, where high levels are associated with low-risk neuroblastoma cases ^1^, was most clearly upregulated in IMR-32 cells ( Figure 2B), in keeping with their mature neuronal morphology following differentiation induction. To further explore the cell types observed in SK-N-BE(2)C cells treated with PB+RA, a selection of human adrenal gland cell type markers were also analysed by qRT-PCR. Strikingly, SK-N-BE(2)C cells displayed significant upregulation of several other markers not observed in IMR-32 or SH-SY5Y cells, including α-smooth muscle actin ( *ACTA2*) associated with myofibroblasts, myosin heavy chain-3 ( *MYH3*) associated with myocytes, phenylethanolamine N-methyltransferase ( *PNMT*) associated with mature adrenal chromaffin cells ^22^ and cytochrome P450 family 11 subfamily A member 1 ( *CYP11A1*) associated with adrenal cortical cells ( Figure 2C, no detectable *CYP11A1* expression in IMR-32 or SH-SY5Y cells). These data suggest ADRN neuroblastoma cell lines show varying responses to PB+RA treatment reminiscent of differentiation into different human adrenal gland cell lineages.

### Differentiation patterns of adrenergic neuroblastoma cell lines correspond to changes in ECM substrate expression

In Figure 6 of 7, RNA-seq analyses were performed on SK-N-BE(2)C cells treated with DMSO vehicle control, PB, RA or PB+RA for five days. This identified a subset of genes (cluster 4) upregulated in cells treated with a combination of PB+RA, but not PB or RA alone ( Figure 1A and C). Gene ontology analyses revealed that these genes were associated with neuronal cell body and extracellular matrix (ECM) cellular components ^7^. Together with our observations in Figure 1 and Figure 2 of this study, this indicated that changes in ECM expression may correspond to differentiation into different human adrenal gland cell lineages. Indeed, Tsokos *et al.* found that specific patterns of ECM expression emerged after differentiation, specifically laminin, fibronectin and collagens, proportional to the normal tissue cell types observed ^8^.

**Figure 3.**
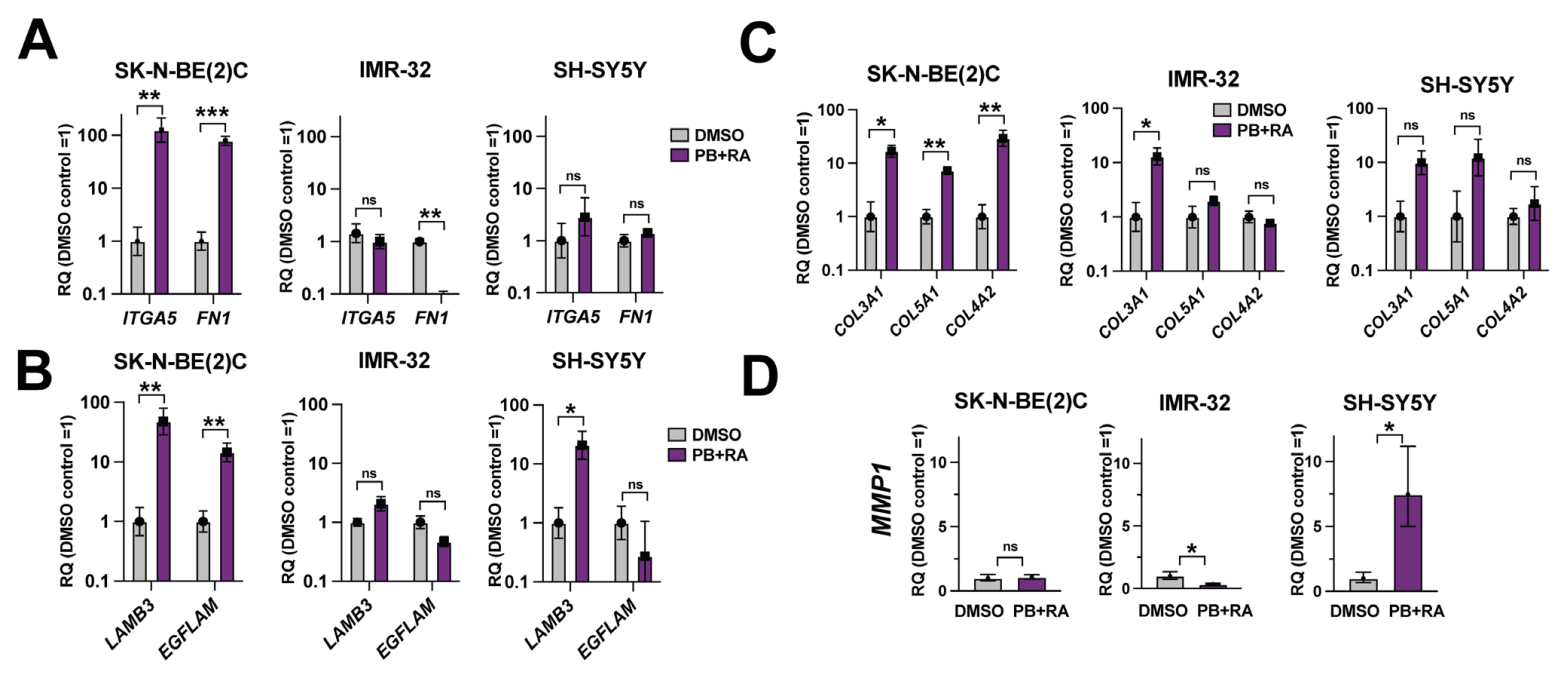
Differentiation patterns of adrenergic neuroblastoma cells correspond to changes in ECM substrate expression. qRT-PCR analysis of ( **A**) *ITGA5* and *FN1*, ( **B**) *LAMB3* and *EGFLAM*, ( **C**) *COL3A1, COL5A1* and *COL4A2* and ( **D**) *MMP1* expression levels in SK-N-BE(2)C, IMR-32 or SH-SY5Y cells treated with DMSO, palbociclib 1 μM (PB), all-trans retinoic acid (10 μM, RA) or PB+RA for 10 days. n = 3 biological replicates. Biological replicates were considered as different passage numbers of the same cell line plated in independent experiments. Mean RQ ± 95% CI. *p ≤ 0.05; **p ≤ 0.01, ***p ≤ 0.001; and ****p ≤ 0.0001. Two-tailed unpaired t-test.

To explore this further, qRT-PCR analyses for selected ECM factors were conducted in SK-N-BE(2)C, IMR-32 and SH-SY5Y cells after 10 days PB+RA treatment, compared to the DMSO vehicle control. These factors were selected from ECM genes upregulated upon PB+RA treatment found in 7. This showed varied patterns of ECM expression in the treated cells. For example, while SK-N-BE(2)C cells showed a significant upregulation in expression of fibronectin ( *FN1*) and the fibronectin receptor Integrin alpha-5 ( *ITGA5)* upon PB+RA treatment, IMR-32 showed a significant downregulation in *FN1* expression ( Figure 3A). Both SK-N-BE(2)C cells and SH-SY5Y showed a clear upregulation in *LAMB3* expression, while SK-N-BE(2)C also showed an upregulation in *EGFLAM*; in comparison IMR-32 showed no significant changes in these factors ( Figure 3B). While all three lines showed an upregulation in *COL3A1*, only SK-N-BE(2)C and SH-SY5Y cells showed upregulation in *COL5A1* and *COL4A2* expression ( Figure 3C). Finally, SH-SY5Y showed a unique upregulation in expression of matrix metalloproteinase 1 ( *MMP1*) upon PB+RA treatment ( Figure 3D). These results suggest that the differentiation responses observed within each cell line are linked to differing expression of ECM factors. 

In conclusion, we present evidence that adrenergic neuroblastoma cells can give rise to mixtures of cell types upon PB+RA-induced differentiation, reminiscent of human adrenal development. We also present data suggesting the varied differentiation responses of each cell line corresponds to changes in patterns of ECM substrate expression. These data build a case to further investigate and consider heterogeneity in neuroblastoma cell differentiation and ECM changes in these cell fate decisions.

## Discussion

Neuroblastoma is a disease of development gone awry, where neural crest-derived progenitor cells become stalled in an immature, proliferative state. Reactivating the latent differentiation potential of these cancer cells is a promising therapeutic approach for this devastating childhood cancer. In this study, we explore the developmental differentiation potential of ADRN-type neuroblastoma cell lines in response to combinatorial treatment with PB+RA, expanding our view of neuroblastoma differentiation beyond neuronal cell fates.

A heterogeneous response to differentiation-induction in neuroblastoma cells was previously reported by Tsokos *et al.* ^8^; three neuroblastoma cell lines, IMR-32, SH-SY5Y and SK-N-SH, were found to undergo three major pathways of differentiation normally followed by differentiating neural crest cells (namely, neuronal, Schwann cell and melanocytic) following combinatorial retinoic acid and dbc-AMP treatment. Our observations are strikingly similar, where IMR-32 cells undergo morphological changes associated with neuronal differentiation, while SH-SY5Y and SK-N-BE(2)C exhibit both neuronal and flat cell phenotypes following PB+RA treatment. Intra-tumoural heterogeneity has been heavily studied in neuroblastoma tumours in recent years, with a differentiation hierarchy found to be similar to that of embryonic adrenal gland development ^23^. While neuroblastoma tumours have been found to resemble proliferative neuroblasts ^1, 24– 26^, the identity of the progenitor cell of origin, the presence of a cancer stem cell hierarchy and variations in the tumour microenvironment could all lead to heterogeneity in cell identity ^23^. Indeed, Jansky *et al.* ^1^ found that, while SK-N-BE(2)C cells showed a higher composition of cycling progenitors, their cell type composition matched states present throughout the adrenal medulla. Treatment with all-trans retinoic acid (RA) reduced the proportion of cycling neuroblasts, however most cells retained progenitor status. While further validation is required, our initial findings suggest combinatorial treatment with both PB and RA could further tip the balance, for some cell lines, towards differentiation into other adrenal gland cell types. We speculate that heterogeneity in differentiation potential intratumorally is associated with heterogeneity in cell identities within the starting tumour; it would be interesting to further investigate the differentiation capacity of a larger cohort of cell lines with I, N and S-type phenotypic characteristics. Interestingly, in PB+RA treated SK-N-BE(2)C cells we observed marker expression outside of the sympathoadrenal lineage, notably ACTA2, MYH3 and CYP11A1. Whilst validation is required, given SK-N-BE(2)C’s characterisation as ‘I-type’ cells this could suggest malignant cells stalled at an early stage of neural crest development result in differentiation into non-malignant mesenchymal cell types. It would indeed be interesting to assess the cell types emerging following differentiation treatment of ‘S-type’ mesenchymal cell lines.

The extracellular matrix is a key component of the microenvironment, providing physical and signalling cues as well as a support scaffold. The role of the ECM during neural crest development is well-described, with cells interacting with a dynamic ECM in a spatiotemporal manner to direct their correct differentiation and migration ^27, 28^. As such, the ECM composition is a key component of many protocols for neuronal differentiation from stem cell states ^29^. The stiffness and rigidity of the ECM also has vital roles in cancer cell biology and migration ^30^. Tsokos *et al.* found specific patterns of ECM expression arose in neuroblastoma cell lines following differentiation, corresponding to the proportions of normal tissue cell types that emerged ^8^. Based on qRT-PCR expression analyses, we observed strikingly similar changes in ECM expression. For example, in fitting with the formation of unattached neurospheres, IMR-32 showed few changes in ECM factor expression upon PB+RA treatment, with exception of a significant downregulation in *FN1* ( Figure 3A). In contrast, a significant increase in *FN1* and *ITGA5* expression was observed in differentiating SK-N-BE(2)C cells; this aligns with differentiation into myofibroblasts (marked by *ACTA2*) which enhance fibronectin assembly ^31^. Myofibroblasts also secrete multiple collagen types, also seen to be significantly upregulated in SK-N-BE(2)C cells following PB+RA treatment ^32^ ( Figure 3C). The unique upregulation of *MMP-1* in SH-SY5Y cells upon PB+RA treatment, a matrix metalloproteinase responsible for degradation of ECM components ^33^, is an interesting observation that may link to unique cell fates in this cell line, noting that high MMP1 expression is traditionally associated with invasive malignancies ^34, 35^

Whether ECM changes during differentiation are a cause or consequence of cell fate decisions remains to be determined and is an interesting topic for further exploration. ECM changes may be a consequence of the emerging cell types, however recent literature suggest ECM can contribute to cell fate decisions in neuroblastoma ^36, 37^. For example, increasing ECM stiffness has been found to modulate neuronal differentiation of neuroblastoma cells ^36^ and ECM topography has been shown to drive a transition between adrenergic and mesenchymal cell types ^37^. It would be interesting to further explore whether ECM modification is able to alter neuroblastoma cell identity in response to PB+RA, and if so, whether drugs altering mechano-transduction pathways may offer additional differentiation benefits. Furthermore, ECM factors secreted by differentiated cells may influence further differentiation of surrounding cells. It will therefore be important to test this in an *in vivo* context, where the cumulative effects of cell types exist. Given the dramatic changes in ECM factor expression, these results also highlight the importance of the tumour microenvironment when modelling neuroblastoma therapy response.

While it remains to be determined how these results translate to an *in vivo* context, these data challenge the notion that neuroblastoma differentiation can only follow the neuronal lineage. Further exploration could include characterisation of the emerging cell types using single-cell RNA-seq, immunocytochemistry and flow cytometry analyses. This would allow an accurate identification and comprehensive characterization of the cell types emerging following PB+RA treatment, as well as the accompanying changes in ECM gene expression; we invite this for further research by the community. Neuroblastomas are graded according to their gangliocytic differentiation state, and it would also be of interest to see if features of other mature neural-crest derived cell types are visible in patient-derived, or mouse modelled, tumour samples following induction of differentiation. We speculate that different tumours, depending on factors such as their cell of origin and microenvironment, may have the capacity to differentiate into varied neural-crest derived cell types, although ADRN cells are dominant and the existence of MES cells is unclear in human tumours ^13^. The differentiation potential of patient-derived cell lines may also be influenced by their genetic background (MYCN amplification and ALK mutation status), the stage of developmental maturation at which progenitors are stalled, intra-tumoral heterogeneity and whether the tumours have undergone prior treatment. Single-cell tracking studies would be insightful in determining whether heterogeneous differentiation occurs in response to treatment-induced plasticity, or is predetermined by the starting cell identity. Finally, it would be of interest to treat cells for extended time periods in vivo, to assess the minimal treatment regime required for stable differentiation. Together, this study builds a case to further investigate and consider heterogeneity in neuroblastoma cell differentiation and ECM changes during these cell fate decisions. Together this study builds a case to further investigate and consider heterogeneity in neuroblastoma cell differentiation and the role of the ECM in these cell fate decisions.

## Ethics and consent statement

Ethical approval and consent were not required.

## Data Availability

The publicly available processed adrenal medulla single cell RNA-seq dataset was used from Jansky *et al.*, 2021 ^1^. European Genome-Phenome Archive (EGA): Neuroblastoma and adrenal gland single-cell study. Accession number: EGAS00001004388; https://ega-archive.org/studies/EGAS00001004388 ^1^. The SK-N-BE(2)C bulk RNA-seq dataset was used from Ferguson & Gillen *et al.*, 2023 ^7^. Gene Expression Omnibus (GEO): Palbociclib releases the latent differentiation capacity of neuroblastoma cells [RNA-seq PB and RA]. Accession number: GSE216274; https://www.ncbi.nlm.nih.gov/geo/query/acc.cgi?acc=GSE216274 ^7^. Zenodo: slgillen/differentiation_response_heterogeneity_transcriptomic_analyses. https://doi.org/10.5281/zenodo.13905809 ^38^. This project contains the following underlying data: Code for the corresponding analysis in Figure 1 of this manuscript. Zenodo: Neuroblastoma cell lines display heterogeneity in differentiation responses. https://doi.org/10.5281/zenodo.16879105 ^39^ This project contains the following underlying data: Underlying data for Figure 2A-C and Figure 3A-D. Figure 2A: Representative phase-contrast images. Figure 2B-C and Figure 3A-D: GraphPad Prism file of raw qRT-PCR ddCt values, Mean RQ ± 95% CI values and statistical analysis. Zenodo: Neuroblastoma cell lines display heterogeneity in differentiation responses. https://doi.org/10.5281/zenodo.16879105 ^39^. This project contains the following extended data: Table S1: qRT-PCR primer sequences, Table S2: List of genes in five clusters identified in 7 and whether they are in single-cell RNA-seq data in 1. Data are available under the terms of the Creative Commons Attribution 4.0 International license (CC-BY 4.0).

## Software availability statement

Archived data analysis code available from: Zenodo - https://doi.org/10.5281/zenodo.13905809 ^38^.

License: Creative Commons Attribution 4.0 International
