# Supplementary material for: Neuroblastoma cell lines display heterogeneity in differentiation responses
Source: Wellcome Open Res. 2025 Sep 16;9:671. Originally published 2024 Nov 12. [Version 2] doi: 10.12688/wellcomeopenres.23249.2 (PMC12676116; doi:10.12688/wellcomeopenres.23249.2)
Supplement: Peer review history

# Reviewer report: Reviewer response for version 2

**DOI:** 10.21956/wellcomeopenres.27411.r140618

**Published:** 2026-01-02

**Authors:** Larisa M Haupt

**Affiliations:** 1 Queensland University of Technology, Brisbane, Australia

The work by Ferguson et al examines the in vitro variability of three neuroblastoma cell lines in response to a combination of differentiation therapies, the CDK4/6 inhibitor palbociclib and retinoic acid. The responses are assessed primarily through gene expression changes via transcription profiling, as well as some morphological features. The work assumes a familiarity both with the team’s previous work as well as the literature surrounding the models used, but primarily their previous work. As such, the manuscript in its current form lacks clarity surrounding the justification of the study as well as the data presented. It is clear the bulk of the data has been presented elsewhere and this manuscript aims to build on this, however, this is difficult to fully interpret without a better justification and summary of the previous data and how this study then augments that data and warrants a follow up study.

 This also extends to the methods section as presented. Details regarding the cell models used as well as the assay design and analysis are needed to more fully understand the study. The figures present some data, however, how was this data selected and why? As to the role of the ECM and/or microenvironment, upregulation of MMP1 does not fully provide an interpretation of this. MMP1 along with other MMPs remodel the local structural components surrounding all cells, in both normal and pathological conditions. It would be helpful to have some clearer justification for the markers validated (?) by Q-PCR as well as the cell lines used to improve the premise of the study would help the reader.

 With three different cell lines used, it is unsurprising differences are observed. The authors made some use of the similarities in the marker profiles identified between the cell lines, but this is difficult to interpret with not enough detail provided on the transcriptome data, the context of the cell models, and some experimental details.

 Some specific comments and questions below:

1. In the introduction, the N/S/I subtypes should be defined. This comes later in the manuscript but it is needed in the introduction to more fully justify the study and the models used.
2. In the Q-PCR data and statistical analysis, it is stated that 3 independent experiments are performed? What does this mean, and how many replicates? It is also stated that there are also biological replicates at different passages? How many, how selected and what is the justification/reasoning for this?
3. SK-N-BE(2)C cells are from a neuroblastoma metastasis, what is the source of the other cells, primary, grade etc vs metastasis – what impact or consideration has this had on the data presented?
4. Are the cell lines inherently heterogeneous? What lineages are represented and in what proportion of the cultures. What proportion of the isolated cells are heterogeneous, does this change with time in culture? What passages were used? How long had the cells been in culture?
5. How long were the cultures maintained/treated and then explored? No details of timepoints are provided.
6. Are SK-N-BE(2)C cells characterised as ‘I-type’ cells? Based on what features? In this study?
7. What is the interest in  mesenchymal cell lines and why? Contribution to the local microenvironment? This is not clear
8. What is the phenotype of the cell cultures, and how was this measured? If it was not measured why not? The proportion of self-renewal vs lineage engaged cells is an important feature of the study.
9. The presentation of the selected RNASeq and Q-PCR data could be more clearly defined and explained.
10. The figures are difficult to interpret effectively, with more details needed in the figure legends as well as the premise of the data summarised in the figures.

Is the work clearly and accurately presented and does it cite the current literature?

Partly

If applicable, is the statistical analysis and its interpretation appropriate?

Partly

Are all the source data underlying the results available to ensure full reproducibility?

Partly

Is the study design appropriate and is the work technically sound?

Partly

Are the conclusions drawn adequately supported by the results?

Partly

Are sufficient details of methods and analysis provided to allow replication by others?

No

Reviewer Expertise:

human neural stem cells, extracellular matrix, human molecular genetics, proteoglycans

I confirm that I have read this submission and believe that I have an appropriate level of expertise to confirm that it is of an acceptable scientific standard, however I have significant reservations, as outlined above.

# Reviewer report: Reviewer response for version 2

**DOI:** 10.21956/wellcomeopenres.27411.r133019

**Published:** 2025-12-03

**Authors:** Sheron Cogo

**Affiliations:** 1 School of Medicine and Health Sciences, Pontifical Catholic University of Parana, Curitiba, Brazil

The authors have satisfactorily addressed all previous comments and clarified the points raised in the first round of review. The revisions have strengthened the manuscript, which presents a clear and robust contribution to the field. I consider the study relevant to the research community and I support its indexing.

Is the work clearly and accurately presented and does it cite the current literature?

Yes

If applicable, is the statistical analysis and its interpretation appropriate?

Yes

Are all the source data underlying the results available to ensure full reproducibility?

Yes

Is the study design appropriate and is the work technically sound?

Partly

Are the conclusions drawn adequately supported by the results?

Partly

Are sufficient details of methods and analysis provided to allow replication by others?

Yes

Reviewer Expertise:

Cell Biology, focused on neuroblastoma in vitro studies.

I confirm that I have read this submission and believe that I have an appropriate level of expertise to confirm that it is of an acceptable scientific standard.

# Reviewer report: Reviewer response for version 2

**DOI:** 10.21956/wellcomeopenres.27411.r133729

**Published:** 2025-10-06

**Authors:** Anand G Patel

**Affiliations:** 1 St. Jude Children’s Research Hospital, , Memphis, Tennessee, USA

In this revised manuscript, Ferguson and colleagues present data analyzing expression and morphologic changes in cell lines treated with retinoic acid and/or palbociclib. Using previously generated bulk transcriptomic data, they analyze 5 gene expression programs. These programs, when overlaid onto reference adrenal development data, map to different cell stages. Microscopy demonstrates morphologic changes in 3 cell lines, and each line has variable transcriptomic changes. Finally, cells treated with both agents showed variable changes in extracellular matrix expression.

 While I think additional experiments – in MES cell lines (to show this effect is specific to ADRN lineage cells) and within in vivo models – would enhance the generalizability of this study, I am aware that this is the second round of review and it would be unfair for me to insist on them.

 However, I do agree with the prior reviewers that relying on morphology and bulk RNA-sequencing continues to be a critical weakness of this study. As the other reviewers commented, the appropriate assay for the question would be single-cell RNA-sequencing. I am sympathetic that this is a difficult and expensive assay, so immunocytochemistry for markers of differentiation would also be acceptable.

Is the work clearly and accurately presented and does it cite the current literature?

Yes

If applicable, is the statistical analysis and its interpretation appropriate?

Yes

Are all the source data underlying the results available to ensure full reproducibility?

Yes

Is the study design appropriate and is the work technically sound?

Yes

Are the conclusions drawn adequately supported by the results?

Partly

Are sufficient details of methods and analysis provided to allow replication by others?

Yes

Reviewer Expertise:

Pediatric oncology, intratumoral heterogeneity

I confirm that I have read this submission and believe that I have an appropriate level of expertise to confirm that it is of an acceptable scientific standard, however I have significant reservations, as outlined above.

# Reviewer report: Reviewer response for version 2

**DOI:** 10.21956/wellcomeopenres.27411.r133731

**Published:** 2025-09-26

**Authors:** Nathaniel W Mabe

**Affiliations:** 1 Purdue University, West Lafayette, Indiana, USA

Ferguson KM and colleagues utilize previously published RNA-sequencing data from SK-N-BE(2)C cells treated with the CDK4/6 inhibitor palbociclib and the differentiation agent retinoic acid alone or in combination to relate gene expression changes to developmental transcriptional programs. The authors assert that differences in gene expression with the combination promote neuroblastic like differentiation programs with elevated expression of ECM components with potential impact on cell fate decisions or therapy. The authors show in three adrenergic-subtype neuroblastoma cell lines that response to ATRA in combination with CDK4/6 inhibition induces differentiation through neurite extensions. This was associated with changes in expression of some ECM-related proteins.

 The manuscript has been extensively reviewed and edited in a prior version (1), and so I will not add significant commentary to reviewers' previous concerns. 

Critiques

 1) gene expression changes from RNA-seq in SK-N-BE(2)C Cells are superimposed onto lineages identified from previous scRNAseq studies. This analysis only shows where significant gene expression programs are reminiscent of developmental trajectories, but this is in no way indicative of cells actually belonging to that differentiation branch. Using a secondary analysis (e.g. western blotting, immunofluorescence) with robust markers that uniquely identify each developmental state is required to assert that they belong to any one of the described states.

 2) The authors allude to different transcriptional programs that are enriched in each cluster (clusters 1-5), but it is not clear what methods were used to describe to these enrichments. Furthermore, a bubbleplot with hypergeometric (overlapping) analysis of enriched gene sets would be helpful.

 3) Cluster 2 is described as being increased in RA only, which is true, but more importantly these genes are blunted by the combination (Which includes RA). Could it be that the blunting of cluster 2 is relevant to observed phenotypes? Cluster 2 is also described as ECM genes according to the description in the text, and thus it is somewhat contradictory to understanding cluster 4 and subsequent discussions around ECM.

 4) After evaluation of the raw data for microscopy images in Figure 2, it is not clear that there is truly differences in morphology between individual treatments and the combination. For example, SKNBE2C and IMR32 with PB and SHSY5Y in DMSO appear to show quite extensive neurites and it is hard to distinguish by eye that there is truly a difference in neurite extensions. Differences interpreted by these micrographs might only be because the cells are lower confluence with the combination relative to other treatment groups. 

 5) The basis for selected ECM factors is unclear. Is there some significance for these genes other than increased from SKNBE2C RNA-seq?

 In conclusion, the study is almost completely reliant on the interpretation of RNA transcription for genes with unknown significance. Heterogeneity in gene expression between cell lines makes interpretation difficult as to whether they have any real significance on the differentiation and/or ECM phenotypes that the authors proposed. Finally, the impact of ECM protein expression and function are not well vetted with complementary experimental approaches (e.g. western blotting, changes in matrix deposition). Thus the interpretation of these data are arbitrary.

Is the work clearly and accurately presented and does it cite the current literature?

Partly

If applicable, is the statistical analysis and its interpretation appropriate?

Partly

Are all the source data underlying the results available to ensure full reproducibility?

Yes

Is the study design appropriate and is the work technically sound?

Partly

Are the conclusions drawn adequately supported by the results?

Partly

Are sufficient details of methods and analysis provided to allow replication by others?

Yes

Reviewer Expertise:

transcriptional/epigenetic plasticity, multi-omics, therapeutics in neuroblastoma

I confirm that I have read this submission and believe that I have an appropriate level of expertise to confirm that it is of an acceptable scientific standard, however I have significant reservations, as outlined above.

# Reviewer report: Reviewer response for version 1

**DOI:** 10.21956/wellcomeopenres.25621.r113580

**Published:** 2024-12-24

**Authors:** Carmen Mejía

**Affiliations:** 1 Universidad Autónoma de Querétaro, Querétaro, Mexico

In the paper called “Neuroblastoma cell lines display heterogeneity in differentiation responses” the authors analyze the response of the NB cell lines SK-N-BE(2)C, IMR32 and SH-SY5Y to the CDK4/6 inhibitor palbociclib (PB), alone or in combination with retinoic acid (RA), a compound that promotes cell differentiation. The objective of this work was to analyze at the gene level using RNAseq how these cell lines differentiate.

 Information on the characteristics of the cell lines is missing.

 They do not show the genes that make up the 5 groups and that is why it is difficult to follow the graphs. I suggest that they include the lists of these genes to know how they contribute to the differentiation analysis.

 From a massive sequencing analysis, it is very risky for the authors to define the lineage that each cell line undergoes as a result of the various treatments.

 In Figure 2, where differentiation is analyzed by confocal microscopy, more information on the different states of differentiation is needed, in addition to quantifying these changes.

 In the same Figure, it is not clear why the authors decided to analyze these genes and not others. It would be important to include something about this in the text.

 It is also not clear whether the authors performed the experiments in triplicate or even a greater number of times, given the statement “3 biological replicates”

 A basal expression control of the cell lines is missing, which would support the response towards differentiation.

 The authors also analyze the expression of ECM factors. This is especially important for cells that acquire a mesenchymal phenotype.

 Finally, they propose studying the heterogeneity of the ECM as a new route in the differentiation process of NB. Something that is not fully supported by the results achieved.

Is the work clearly and accurately presented and does it cite the current literature?

Yes

If applicable, is the statistical analysis and its interpretation appropriate?

Yes

Are all the source data underlying the results available to ensure full reproducibility?

Partly

Is the study design appropriate and is the work technically sound?

Yes

Are the conclusions drawn adequately supported by the results?

Partly

Are sufficient details of methods and analysis provided to allow replication by others?

Partly

Reviewer Expertise:

Childhood oncology, apoptosis, molecular biology, cellular biology.

I confirm that I have read this submission and believe that I have an appropriate level of expertise to confirm that it is of an acceptable scientific standard, however I have significant reservations, as outlined above.

# Response

**Published:** 2025-08-24

**Authors:** Anna Philpott

**Affiliations:** Cambridge Stem Cell Institute, Cambridge, England, UK

We thank the reviewer for their helpful comments and respond to each of these below.

- **Comment 1: ** Information on the characteristics of the cell lines is missing.
- **Author response**:  Thank you for this comment. We have now included available cell line details in the text with references (Results, paragraph 2), including details of phenotypic classifications.

 

- **Comment 2: **They do not show the genes that make up the 5 groups and that is why it is difficult to follow the graphs. I suggest that they include the lists of these genes to know how they contribute to the differentiation analysis.
- **Author response:**  We provided the requested table of genes associated with each signature in the data and code available on zenodo, but we have now added this to Extended Data, Table S2, so that it is more readily findable, and have directly referred to this table within the text and figure legend. In addition, as often a greater number of genes are detectable by bulk RNA-seq than scRNA-seq, we have added a column to this table 'gene_in_Jansky_et_al_2021' to be clear which genes from our clusters derived from bulk RNA-seq were also detectable in the single cell RNA-seq dataset. We have now also clarified in the legend of Figure 1A the gene groups associated with each cluster 1-5 (identified in our previous publication [7]).

 

- **Comment 3**: From a massive sequencing analysis, it is very risky for the authors to define the lineage that each cell line undergoes as a result of the various treatments…..
- **Author response: ** We believe we have been cautious in not over-defining the lineages or exact cell types emerging. We have presented the findings in Figure 1 with supporting qRTPCR data, presenting interesting gene expression changes reminiscent of differentiation into different human adrenal gland cell lineages. We are aware of the caveats of bulk sequencing analyses, and indeed agree in the discussion that single-cell analysis would be enriching (Discussion, paragraph 5). However, despite our analyses being at the bulk level we see significant gene expression changes, worthy of presenting as interesting data for future exploration in the field of neuroblastoma differentiation heterogeneity.

 

- **Comment 4:** In Figure 2, where differentiation is analyzed by confocal microscopy, more information on the different states of differentiation is needed, in addition to quantifying these changes.
- **Author response:**  We agree with the Reviewer that further exploration of the cell types emerging from PB+RA treatment of each cell line could be performed. As stated in the discussion ‘Further exploration could include characterisation of the emerging cell types using single-cell RNA-seq, immunocytochemistry and flow cytometry analyses.’ It would have been ideal to perform a full characterisation of the emerging cell types, using single cell techniques such as single cell RNA-seq.  This would allow an accurate identification and comprehensive characterization of the cells emerging following PB+RA treatment, using the markers identified for validation by ICC and flow cytometry. However, this is unfortunately not possible due to funding constraints. We have expanded on these future work suggestions in the discussion and invite this for further research (Discussion, paragraph 5).

 

- **Comment 5:** In the same Figure, it is not clear why the authors decided to analyze these genes and not others. It would be important to include something about this in the text.
- **Author response: ** The differentiation markers shown in Figure 2 were selected using marker genes of adrenal medullary cell populations referenced in Jansky et al [1].  NEFM, GAP43 and SYT5 were chosen as markers of sympathetic neuroblasts. PNMT, the enzyme catalyzing methylation of norepinephrine to form epinephrine, was chosen as a marker of mature chromaffin cells, ACTA2 as a marker of myofibroblasts, MYH3 as a marker of myocytes, and CYP11A1 as a marker of adrenal cortical cells. This has been clarified in the text of the Results (section 2). We note that CYP11A1, as well as MYH3 and ACTA2, mark populations outside of the sympathoadrenal lineage - this is indeed interesting and could suggest malignant transformation at an early stage of neural crest development from bipotent or mesenchymal lineage precursors, and/or plasticity of neuroblastoma cells (26) - we have discussed this further in the text (Discussion, paragraph 2). We agree that it would have been ideal to perform a full characterisation of the emerging cell types, encompassing all cell type markers, ECM factors, and transcription factors in Jansky et al [1] and more, using single cell RNA-seq - we have expanded on these future work suggestions in the discussion and invite this for further research (Discussion, paragraph 5).

 

- **Comment 6: **It is also not clear whether the authors performed the experiments in triplicate or even a greater number of times, given the statement “3 biological replicates”
- **Author response:  **Three biological replicates were performed for each cell line, corresponding to three independent passages of each cell line. As stated in the ‘Statistical Analysis’ section of the Methods, ‘Biological replicates were considered as different passage numbers of the same cell line plated in independent experiments’. This has been further clarified in the text of the figure legends for Figures 2 and 3.

 

- **Comment 7:** A basal expression control of the cell lines is missing, which would support the response towards differentiation.
- **Author response:**  Regarding the comparison of basal levels, the experiments were performed in a manner allowing us to compare DMSO with PB+RA treatment within each cell line. We therefore make conclusions as to whether fold changes in gene expression are seen within each line in response to PB+RA treatment. Comparison of the basal gene expression levels would indeed be also interesting, however is beyond the scope of the manuscript.

 

- **Comment 8:** The authors also analyze the expression of ECM factors. This is especially important for cells that acquire a mesenchymal phenotype.
- **Author response:**  We agree with the Reviewer that expression of ECM factors may be particularly pertinent to differentiation into non-neuronal lineages. As discussed in the text (Results, section 3), our investigations into ECM factor expression were led by Tsokos et al’s finding [8] that “specific patterns of ECM expression emerged after differentiation, proportional to the normal tissue cell types observed”, particularly laminin, fibronectin and collagen. Our choice of ECM markers was guided by this list of genes, found to be differentially upregulated in PB+RA treated cells in [7]. We have now clarified this in the text (Results, section 3). We also mention in the discussion how ECM factors relate to mesenchymal-type differentiation - “For example, in fitting with the formation of unattached neurospheres, IMR-32 showed few changes in ECM factor expression upon PB+RA treatment, with exception of a significant downregulation in FN1 (Figure 3A). In contrast, a significant increase in FN1 and ITGA5 expression was observed in differentiating SK-N-BE(2)C cells; this aligns with differentiation into myofibroblasts (marked by ACTA2) which enhance fibronectin assembly. Myofibroblasts also secrete multiple collagen types, also seen to be significantly upregulated in SK-N-BE(2)C cells following PB+RA treatment”.

 

- **Comment 9**: Finally, they propose studying the heterogeneity of the ECM as a new route in the differentiation process of NB. Something that is not fully supported by the results achieved.
- **Author response**:  As in Tsokos et al [8], our data support that differentiation responses correspond to changes in patterns of ECM substrate expression. We agree with the Reviewer that whether changes in ECM substrate expression are a cause or a consequence of differentiation remains to be determined.

As noted, there are indications in the literature that the ECM may direct cell fate choices, and for example reference 36 where increasing ECM stiffness has been found to modulate neuronal differentiation of neuroblastoma cells and reference 37 where ECM topography has been shown to drive a transition between adrenergic and mesenchymal cell types. As noted, ‘The role of the ECM during neural crest development is well-described, with cells interacting with a dynamic ECM in a spatiotemporal manner to direct their correct differentiation and migration. As such, the ECM composition is a key component of many protocols for neuronal differentiation from stem cell states [29]’. In referencing this literature in the first manuscript version, we intended to discuss whether ECM changes are a cause or consequence of cell fate decisions as an interesting topic for further exploration. For example, ‘it would be interesting to further explore whether ECM modification is able to alter neuroblastoma cell identity in response to PB+RA, and if so, whether drugs altering mechano-transduction pathways may offer additional differentiation benefits.’ We have added to the text to clarify this (Discussion, Paragraph 4), acknowledging that our data are an initial observation to prompt further investigation. It would be important to test this in an in vivo system where cumulative effects of cell types will exist, and have further discussed this in the discussion in paragraph 4. We have also modified the text from ‘build a case… for investigating the effect of ECM on neuroblastoma cell fate decisions’ to ‘investigating ECM changes in neuroblastoma differentiation’ (e.g. end of Introduction and end of Results) to reflect this increased discussion.   We thank the reviewer for their helpful comments which have guided submission of version 2 of this manuscript. While we note that the Reviewer quite rightly states that we could take this work further, we are unable to do so because of funding and staffing constraints.  However, we feel strongly that it is important to disseminate our findings to allow others to build on the work that we have done and feel that Wellcome Open Research would be an appropriate venue for this. We hope that the reviewer can consider this when evaluating our responses.

# Reviewer report: Reviewer response for version 1

**DOI:** 10.21956/wellcomeopenres.25621.r113577

**Published:** 2024-12-23

**Authors:** Sheron Cogo

**Affiliations:** 1 School of Medicine and Health Sciences, Pontifical Catholic University of Parana, Curitiba, Brazil

This study investigates the heterogeneity in differentiation responses of three adrenergic-type (ADRN) neuroblastoma cell lines: SK-N-BE(2)C, IMR-32, and SH-SY5Y, treated with a combination of retinoic acid (RA) and palbociclib (PB), a CDK4/6 inhibitor. The research extends previous findings demonstrating the potential of PB-RA combination to promote neuronal differentiation in ADRN cells.

 According to the authors, the combination of PB and RA induced cell differentiation in ADRN cells, and reduced proliferation, broadening the understanding of differentiation therapy. Integration of morphological, transcriptomic, and qRT-PCR analyses provided a comprehensive view of cellular responses to the treatment.

 They demonstrate diverse phenotypic responses and transcriptomic alterations, in which, according to the authors, IMR-32 predominantly differentiated into neurons, while SK-N-BE(2)C and SH-SY5Y showed differentiation into other cell types, including flattened phenotypes, suggesting non-neuronal differentiation trajectories. However, divergent results among ADRN cell lines, or N-type phenotypes, could also be associated to cross-contamination, genetic drift or even phenotypic changes caused by prolonged passages. Therefore, authentication through STR profiling or similar methods should be highly recommended.

 S-type and I-type cells exhibit greater plasticity, being able to transition between different cellular states (Campos Cogo et al., 2020). These cell lines could differ in both the extent and type of differentiation induced by PB-RA treatment. Some cell lines in the study are derived from other cell lines; the SH-SY5Y cell line, for example, is the N-phenotype derived from SK-N-SH, an I-phenotype; and it has been described that both maintain the capacity for trans differentiation, associated with epithelial-mesenchymal transitions (Gautier et al., 2021). Highlighting the importance of explaining the reasons behind the selection of lines for the purpose of the study and reinforces the need to authenticate the cell lines.

 Also, regarding the characterization of differentiation processes, the light microscopy images presented are not sufficient to demonstrate the morphological differences associated with the differentiation processes, especially since they reflect cultures in different states of confluence. It would be interesting to use specific fluorescent markers for different differentiation phenotypes that could be quantified. Methods like immunofluorescence or flow cytometry could validate the emerging cell types. Furthermore, could also help to clearly distinguish between true cellular differentiation and cellular plasticity, which is essential for interpreting the results.

 RNA-Seq of SK-N-BE(2)C identified gene signatures associated with differentiation trajectories into various adrenal gland lineages and qRT-PCR analysis revealed distinct gene expression patterns across all cell lines, suggesting intrinsic heterogeneity or alterations in the cellular microenvironment. The use of bulk RNA-Seq, however, limits the ability to identify cellular-level heterogeneity. If possible, single-cell RNA-Seq data could confirm distinct differentiation trajectories.

 Genes related to extracellular matrix (ECM) were differentially regulated in response to PB-RA treatment. Although, it remains unclear whether ECM changes are a cause or consequence of differentiation trajectories. Detailed mechanistic explorations of ECM’s role in modulating cellular differentiation and the rationale for the chosen genes should be better contextualized in the study. Including additional genes associated with cellular plasticity or epithelial-to-mesenchymal transition could enrich the findings.

 Regarding the incubation period with RA, its determination has not been clearly explained. Five days of RA incubation may be insufficient to observe mature phenotypic changes. Most studies with SH-SY5Y differentiation, for example, use 7-14 days of incubation (Cheung et al., 2009; Kartal & Arslan Yildiz, 2024; Shipley et al., 2016). Future studies could extend treatment periods (7–14 days) to better capture cellular responses.

 This study provides significant insights into the heterogeneity of differentiation responses in neuroblastoma cells. However, experimental limitations, such as the lack of single-cell data and cell line authentication, restrict some conclusions. With refinements, this research has the potential to offer more robust insights into the interaction between ECM and cellular differentiation in neuroblastoma tumors.

Is the work clearly and accurately presented and does it cite the current literature?

Yes

If applicable, is the statistical analysis and its interpretation appropriate?

Yes

Are all the source data underlying the results available to ensure full reproducibility?

Yes

Is the study design appropriate and is the work technically sound?

Partly

Are the conclusions drawn adequately supported by the results?

Partly

Are sufficient details of methods and analysis provided to allow replication by others?

Yes

Reviewer Expertise:

Cell Biology, focused on neuroblastoma in vitro studies.

I confirm that I have read this submission and believe that I have an appropriate level of expertise to confirm that it is of an acceptable scientific standard, however I have significant reservations, as outlined above.

## References

[1] : An overview of neuroblastoma cell lineage phenotypes and in vitro models. *Exp Biol Med (Maywood)* .2020;245(18) : 10.1177/1535370220949237 1637-1647 10.1177/1535370220949237 32787463 PMC7802384

[2] : Effects of all-trans-retinoic acid on human SH-SY5Y neuroblastoma as in vitro model in neurotoxicity research. *Neurotoxicology* .2009;30(1) : 10.1016/j.neuro.2008.11.001 127-35 10.1016/j.neuro.2008.11.001 19056420

[3] : Exploring Neuronal Differentiation Profiles in SH-SY5Y Cells through Magnetic Levitation Analysis. *ACS Omega* .2024;9(13) : 10.1021/acsomega.3c08962 14955-14962 10.1021/acsomega.3c08962 38585102 PMC10993277

[4] : Differentiation of the SH-SY5Y Human Neuroblastoma Cell Line. *J Vis Exp* .2016; 10.3791/53193 53193 10.3791/53193 26967710 PMC4828168

[5] : Plasticity in Neuroblastoma Cell Identity Defines a Noradrenergic-to-Mesenchymal Transition (NMT). *Cancers (Basel)* .2021;13(12) : 10.3390/cancers13122904 10.3390/cancers13122904 34200747 PMC8230375

# Response

**Published:** 2025-08-24

**Authors:** Anna Philpott

**Affiliations:** Cambridge Stem Cell Institute, Cambridge, England, UK

We thank the reviewer for their helpful comments and respond to each of these below.

- **Comment 1: **They demonstrate diverse phenotypic responses and transcriptomic alterations, in which, according to the authors, IMR-32 predominantly differentiated into neurons, while SK-N-BE(2)C and SH-SY5Y showed differentiation into other cell types, including flattened phenotypes, suggesting non-neuronal differentiation trajectories. However, divergent results among ADRN cell lines, or N-type phenotypes, could also be associated to cross-contamination, genetic drift or even phenotypic changes caused by prolonged passages. Therefore, authentication through STR profiling or similar methods should be highly recommended.
- **Author response:** We can confirm for the Reviewer that our cell lines are regularly STR tested. We therefore do not have reason to believe the divergent results we observe are due to genetic drift across passages or cross-contamination.

 

- **Comment 2:** S-type and I-type cells exhibit greater plasticity, being able to transition between different cellular states (Campos Cogo et al., 2020). These cell lines could differ in both the extent and type of differentiation induced by PB-RA treatment. Some cell lines in the study are derived from other cell lines; the SH-SY5Y cell line, for example, is the N-phenotype derived from SK-N-SH, an I-phenotype; and it has been described that both maintain the capacity for trans differentiation, associated with epithelial-mesenchymal transitions (Gautier et al., 2021). Highlighting the importance of explaining the reasons behind the selection of lines for the purpose of the study and reinforces the need to authenticate the cell lines.
- **Author response:** Thank you for this comment. We have now included available cell line details in the text with references (Results, section 2), including details of phenotypic classifications. We have also added to the discussion (Discussion, paragraph 2), highlighting the link between initial cell line heterogeneity and differentiation potential is interesting for exploration in a larger cohort of cell lines representing I, N and S-types.

 

- **Comment 3:** Also, regarding the characterization of differentiation processes, the light microscopy images presented are not sufficient to demonstrate the morphological differences associated with the differentiation processes, especially since they reflect cultures in different states of confluence. It would be interesting to use specific fluorescent markers for different differentiation phenotypes that could be quantified. Methods like immunofluorescence or flow cytometry could validate the emerging cell types.
- **Author response**: We agree with the Reviewer that further exploration of the cell types emerging from PB+RA treatment of each cell line could be performed. As stated in the discussion ‘Further exploration could include characterisation of the emerging cell types using single-cell RNA-seq, immunocytochemistry and flow cytometry analyses.’ It would have been ideal to perform a full characterisation of the emerging cell types, using single cell techniques such as single cell RNA-seq. This would allow an accurate identification and comprehensive characterization of the cells emerging following PB+RA treatment, using the markers identified. However, this is unfortunately not possible due to funding and staffing constraints. We have expanded on these future work suggestions in the discussion and invite this for further research (Discussion, paragraph 5). Regarding the differentiation images, additional low-confluence images were not provided as these experiments are a follow-on from previous studies where live imaging is performed to track the cells from D0 of treatment [7]. These movies show clearly the morphology of the cells prior to treatment, which we have clarified in the text for the readers’ reference (Figure 2 legend).

 

- **Comment 4: **Furthermore, could also help to clearly distinguish between true cellular differentiation and cellular plasticity, which is essential for interpreting the results.
- **Author response:** Thank you for the discussion regarding plasticity vs differentiation. In all three cell lines tested, we observe a dramatic decrease in proliferation with PB+RA treatment (seen in Figures 5, S4, S6, 7 and videos of [7]), further evident after 10 days. This is in contrast to the highly proliferative nature of MES-type neuroblastoma cell lines, such as SH-EP, which may be expected if cells were simply interconverting between malignant cell states. It would indeed be interesting to determine if the resulting cell states seen after treatment are stably differentiated, and single-cell RNA-seq to determine markers of the emerging cell types would be extremely useful to resolve this question. Such an experiment is difficult to truly determine in vitro however; we therefore suggest in vivo experiments would be required to assess the terminal differentiation of the resulting cell states, which is beyond the scope of this manuscript. We have now noted the literature on cell line plasticity in the Introduction (paragraph 2) and discuss that phenotypic heterogeneity in cell lines may influence differentiation capacity. Furthermore, we have added to the Discussion (Discussion, paragraph 5) - “Single-cell tracking studies would be insightful to determine if heterogeneous differentiation occurs in response to treatment-induced plasticity, or is predetermined by starting cell identity. Finally, it would also be of interest to treat cells for further extended time periods, in vitro and in vivo, as well as investigate whether continued treatment is required for stable differentiation”.

 

- **Comment 5**: RNA-Seq of SK-N-BE(2)C identified gene signatures associated with differentiation trajectories into various adrenal gland lineages and qRT-PCR analysis revealed distinct gene expression patterns across all cell lines, suggesting intrinsic heterogeneity or alterations in the cellular microenvironment. The use of bulk RNA-Seq, however, limits the ability to identify cellular-level heterogeneity. If possible, single-cell RNA-Seq data could confirm distinct differentiation trajectories.
- **Author response:** We agree with the Reviewer that further exploration of the cell types emerging from PB+RA treatment of each cell line could be performed. As stated in the discussion ‘Further exploration could include characterisation of the emerging cell types using single-cell RNA-seq, immunocytochemistry and flow cytometry analyses.’ It would have been ideal to perform a full characterisation of the emerging cell types, using single cell techniques such as single cell RNA-seq. This would allow an accurate identification and comprehensive characterization of the cells emerging following PB+RA treatment, using the markers identified for validation by ICC and flow cytometry, however, this is unfortunately not possible. We have expanded on these future work suggestions in the discussion and invite this for further research (Discussion, paragraph 5).

 

- **Comment 6:** Genes related to extracellular matrix (ECM) were differentially regulated in response to PB-RA treatment. Although, it remains unclear whether ECM changes are a cause or consequence of differentiation trajectories. Detailed mechanistic explorations of ECM’s role in modulating cellular differentiation and the rationale for the chosen genes should be better contextualized in the study. Including additional genes associated with cellular plasticity or epithelial-to-mesenchymal transition could enrich the findings.
- **Author response:** We thank the Reviewer for this insightful comment. As in Tsokos et al [8], our data support that differentiation responses correspond to changes in patterns of ECM substrate expression. We agree with the Reviewer that whether changes in ECM substrate expression are a cause or a consequence of differentiation remains to be determined.

As noted, there are indications in the literature that the ECM may direct cell fate choices, and for example reference 36 where increasing ECM stiffness has been found to modulate neuronal differentiation of neuroblastoma cells and reference 37 where ECM topography has been shown to drive a transition between adrenergic and mesenchymal cell types. As noted, ‘The role of the ECM during neural crest development is well-described, with cells interacting with a dynamic ECM in a spatiotemporal manner to direct their correct differentiation and migration. As such, the ECM composition is a key component of many protocols for neuronal differentiation from stem cell states [29]’. In referencing this literature in the first manuscript version, we intended to discuss whether ECM changes are a cause or consequence of cell fate decisions as an interesting topic for further exploration. For example, ‘it would be interesting to further explore whether ECM modification is able to alter neuroblastoma cell identity in response to PB+RA, and if so, whether drugs altering mechano-transduction pathways may offer additional differentiation benefits.’ We have added to the text to clarify this (Discussion, Paragraph 4), acknowledging that our data are an initial observation to prompt further investigation. It would be important to test this in an in vivo system where cumulative effects of cell types will exist, and have further discussed this in the discussion in paragraph 4. We have also modified the text from ‘build a case… for investigating the effect of ECM on neuroblastoma cell fate decisions’ to ‘investigating ECM changes in neuroblastoma differentiation’ (e.g. end of Introduction and end of Results) to reflect this increased discussion. Regarding our choice of ECM factors, as discussed in the Results (section “Differentiation patterns of adrenergic neuroblastoma cell lines correspond to changes in ECM substrate expression“) our investigations into ECM factor expression were led by Tsokos et al’s finding [8] that “specific patterns of ECM expression emerged after differentiation, proportional to the normal tissue cell types observed”, particularly laminin, fibronectin and collagen. In addition, “RNA-seq analyses were performed on SK-N-BE(2)C cells treated with DMSO vehicle control, PB, RA or PB+RA for five days [7]. This identified a subset of genes (cluster 4) upregulated in cells treated with a combination of PB+RA, but not PB or RA alone (Figure 1A and C). Gene ontology analyses revealed that these genes were associated with neuronal cell body and extracellular matrix (ECM) cellular components”. Our choice of ECM markers was guided by Tsokos et al and these genes found to be differentially upregulated in PB+RA treated cells. We have now clarified this in the Results text (paragraph 4-5). As mentioned ‘Further exploration could include characterisation of the emerging cell types using single-cell RNA-seq, immunocytochemistry and flow cytometry analyses.’ As well as the emerging cell states, this would enable a full characterisation of ECM factors and their relation to individual cell types, as well as other markers of plasticity and EMT mentioned by the reviewer. Unfortunately, this wide-scale characterization was not possible due to time and funding constraints; we have added discussion about this as a future area of research (paragraph 5).  

- **Comment 7:** Regarding the incubation period with RA, its determination has not been clearly explained. Five days of RA incubation may be insufficient to observe mature phenotypic changes. Most studies with SH-SY5Y differentiation, for example, use 7-14 days of incubation (Cheung et al., 2009; Kartal & Arslan Yildiz, 2024; Shipley et al., 2016). Future studies could extend treatment periods (7–14 days) to better capture cellular responses.
- **Author response:** In this study, we lengthened the treatment of cells from 5 days to 10 days with PB+RA to further investigate the differentiation responses between cell lines. We agree that it would be interesting to treat cells for even longer time periods, in vitro or in vivo, as well as investigate if continued treatment is required for stable differentiation. We have added this as possible future work to the discussion text (Discussion, paragraph 5).

    We thank the reviewer for their helpful comments which have guided submission of version 2 of this manuscript. While we note that the Reviewer quite rightly states that we could take this work further, we are unable to do so because of staffing constraints. However, we feel strongly that it is important to disseminate our findings to allow others to build on the work that we have done, and feel that Wellcome Open Research is an appropriate venue for this. We hope that the reviewer can consider this when evaluating our responses.

# Reviewer report: Reviewer response for version 1

**DOI:** 10.21956/wellcomeopenres.25621.r113578

**Published:** 2024-12-19

**Authors:** Cécile Thirant

**Affiliations:** 1 Institut Curie, Paris, France

This research article entitled *“Neuroblastoma cell lines display heterogeneity in differentiation responses” *by Ferguson KM *et al.*, addresses the *in vitro* variability of morphological and transcriptional responses of three neuroblastoma (NB) cell lines to a combo of differentiation therapies using palbociclib (a CDK4/6 inhibitor, PB) and retinoic acid (RA).

 This work is based on and aims to extend the data produced by the same authors in the recent Ferguson KM *et al.*, in Developmental Cell, 2023. In this paper, they published epigenetic and transcriptomic data (using RNAseq) as well as morphological assays of the SK-BE(2)C cell line treated 5 days +/- PB and RA pointing to an epigenetic rewiring associated to a differentiation effect with neurite extensions and the identification of 5 clusters of deregulated genes. In this new study, the authors are interested in the potential of differentiation of NB cell lines induced by PB+RA.

 1. In the introduction, the authors may reformulate these two sentences: *“During normal development, sympathoadrenal precursor cells derived from the neural crest differentiate into cell types including sympathetic neurons, adrenal chromaffin cells and Schwann cells. In neuroblastoma, these sympathetic precursor cells fail to differentiate and are locked into an immature state that drives tumour growth”.* First, they are a copy and paste from Ferguson KM DevCell 2023 introduction and then, to my knowledge, neural crest cells indeed produced neurons, chromaffins and Schwann cells, but sympathoadrenal progenitors do not produce Schwann cells.

 2. In the results, the authors re-used their RNAseq data obtained in the SKNBE2C cell line. They projected the deregulated genes upon treatment at bulk level into the trajectory of differentiation drawn from scRNAseq studies on normal adrenal development, published by Jansky *et al*. They show that almost all clusters could be highlighted by one of the lists of genes, and propose that this cell line has the potential to enter different lineage pathways, in addition to neuronal differentiation.

 Figure 1C: Could the authors harmonize the scale and avoid -0.05. I think it would be helpful to provide as supplementary tables the lists of genes used to define the 5 clusters and plotted as signatures. Could the authors also indicate how many and which genes from these lists contribute to the plotted signatures ?

 However, without any single-cell RNAseq data showing that after treatment, distinct cells harbor distinct cell type genes according to normal differentiation, it is really difficult from bulk RNAseq data to suggest that those cells “have the potential to enter different human adrenal medullary developmental lineages (…).”

 3. The study follows by the more precise characterization of the heterogeneity of differentiation response to PB+RA of distinct cell lines, using 3 neuroblastoma models and a prolonged exposure of treatment of 10 days.

 First, in addition to their distinct genetic background, could the authors precise the different origin of the three selected NB models ? Primary tumor or metastases; before or after treatment; grade; primary tumor location (adrenal gland or sympathetic ganglia) etc… As neuroblastoma is a disease well known for its heterogeneity and so possibly “stalled at different stages of developmental differentiation” as rightly mentioned by the authors in the introduction, could this point be discussed because possibly explaining the obtained results?

 Minor point: “These cell lines represent a mixture of genetic background” should be replace by “These cell lines represent different genetic backgrounds”.

 4. To illustrate distinct differentiation states after treatment, they looked at the cellular morphology (Figure 2A). Whereas the IMR-32 cell line seems only differentiating towards a neuronal fate, BE2C and 5Y showed other phenotype that is not easy to recognize using phase-contrast images. Could the authors find a way to quantify the changes of morphology ? IF maybe ? Photos of control cells at confluence do not allow to really evaluate changes of morphology and may be replace.

 5. Additionally, they looked at the expression of selected genes by RT-qPCR to evaluate the potential of differentiation in distinct normal cell types of the sympathetic nervous system of the tumor cell lines treated with PB+RA.

 - How these genes have been selected ? It is quite difficult to be certain of a way of differentiation based on the expression of one marker, and at the bulk level. Of note, adrenal cortical cells are coming from a complete different tissue of origin than the adrenal medulla or the sympathetic ganglia, so could the authors justify the use of *CYP11A1* ?

 - How many times this experiment has been conducted ? Does “3 biological replicates” mean 3 independent experiments with how many technical replicates ? To better compare responses between cell lines and to conclude about inter-cell line heterogeneity, it would be necessary to compare also their basal level of expression.

 6. Whereas the IMR32 and the 5Y seem mainly able to go towards a neuronal differentiation, the BE2C seems able to express genes of several cell types from adrenergic to mesenchymal phenotypes (ACTA2, ITGA5, FN1, COLs etc…). Interestingly this cell line has been previously used to study NB tumor cell plasticity, cells being able to shift from a noradrenergic towards a mesenchymal identity. Why the authors did not address the summary of literature on this subject or at least discuss this point? When cells after treatment change phenotypes, in what degree can we distinguish differentiation versus plasticity ?

 7. Finally, authors looked at ECM factors expression, which is inherently linked to cell identity (especially for mesenchymal cells). Again, the pertinence of looking to these particular genes is missing and would help to interpret the data.

 What is the biological difference between COL3A1, COL5A1 and COL4A2? Is it known that each of this gene is associated to a specific cell type? Same question about LAMB3 and EGFLAM, why the authors selected those genes?

 8. Concerning the conclusion, these results illustrate inter- neuroblastoma cell line heterogeneity of responses to this therapy, in line with the fact that heterogeneity is a well-known characteristic of neuroblastoma. However, pointing towards “ the role of the ECM in these cell fate decisions” is over-interpreted, not shown nor suggested here, as I would rather say that ECM factors here reflect tumor cell identity and not the other way round.

 Minor point: does this journal accept references of biorXiv papers (ref 26)?

Is the work clearly and accurately presented and does it cite the current literature?

Partly

If applicable, is the statistical analysis and its interpretation appropriate?

Partly

Are all the source data underlying the results available to ensure full reproducibility?

Partly

Is the study design appropriate and is the work technically sound?

Partly

Are the conclusions drawn adequately supported by the results?

Partly

Are sufficient details of methods and analysis provided to allow replication by others?

Yes

Reviewer Expertise:

Pediatric cancers including Neuoblastoma tumor cell plasticity and heterogeneity

I confirm that I have read this submission and believe that I have an appropriate level of expertise to confirm that it is of an acceptable scientific standard, however I have significant reservations, as outlined above.

# Response

**Published:** 2025-08-24

**Authors:** Anna Philpott

**Affiliations:** Cambridge Stem Cell Institute, Cambridge, England, UK

We thank the reviewer for their helpful comments and respond to each of these below.

- **Comment 1:** In the introduction, the authors may reformulate these two sentences: “During normal development, sympathoadrenal precursor cells derived from the neural crest differentiate into cell types including sympathetic neurons, adrenal chromaffin cells and Schwann cells. In neuroblastoma, these sympathetic precursor cells fail to differentiate and are locked into an immature state that drives tumour growth”. First, they are a copy and paste from Ferguson KM DevCell 2023 introduction and then, to my knowledge, neural crest cells indeed produced neurons, chromaffins and Schwann cells, but sympathoadrenal progenitors do not produce Schwann cells.
- **Author response**: Thank you for bringing this to our attention. We have reworded the above introductory sentences and have amended the text (paragraph 1, Introduction).

   

- **Comment 2a**: In the results, the authors re-used their RNAseq data obtained in the SKNBE2C cell line. They projected the deregulated genes upon treatment at bulk level into the trajectory of differentiation drawn from scRNAseq studies on normal adrenal development, published by Jansky et al. They show that almost all clusters could be highlighted by one of the lists of genes, and propose that this cell line has the potential to enter different lineage pathways, in addition to neuronal differentiation. Figure 1C: Could the authors harmonize the scale and avoid -0.05. I think it would be helpful to provide as supplementary tables the lists of genes used to define the 5 clusters and plotted as signatures. Could the authors also indicate how many and which genes from these lists contribute to the plotted signatures ?
- **Author response:** In Figure 1C the values and scale represent a module score for a given geneset. This was conducted using Seurat AddModuleScore from which the scores reflect how the expression of a given gene set varies across the single cells in the dataset compared to a randomly selected set of control genes. Therefore, it is most appropriate for the scales to remain as originally plotted for each plot. We provided the requested table of genes associated with each signature in the data and code available on zenodo, but we have now added this to Extended Data, Table S2, so that it is more readily findable, and have directly referred to this table within the text and figure legend. In addition, as often a greater number of genes are detectable by bulk RNA-seq than scRNA-seq, we have added a column to this table 'gene_in_Jansky_et_al_2021' to be clear which genes from our clusters derived from bulk RNA-seq were also detectable in the single cell RNA-seq dataset. We have now also clarified in the Figure 1 legend and Results text the gene groups associated with each cluster 1-5 (identified in our previous publication [7]).

 

- **Comment 2b**: However, without any single-cell RNAseq data showing that after treatment, distinct cells harbor distinct cell type genes according to normal differentiation, it is really difficult from bulk RNAseq data to suggest that those cells “have the potential to enter different human adrenal medullary developmental lineages (…).”
- **Author response:** We agree with the Reviewer that further exploration of the cell types emerging from PB+RA treatment of each cell line could be performed. As stated in the discussion ‘Further exploration could include characterisation of the emerging cell types using single-cell RNA-seq, immunocytochemistry and flow cytometry analyses.’ We would very much like to perform a full characterisation of the emerging cell types, using single cell techniques - this would allow an accurate identification and comprehensive characterization of the cells emerging following PB+RA treatment. However, this is unfortunately not possible due to funding and staffing constraints. We have expanded on these future work suggestions in the discussion and invite this for further research (Discussion, paragraph 5). We have also modified the text to ‘While further validation is required as detailed in the discussion…this suggests that SK-N-BE(2)C neuroblastoma cells may have the potential…’ (Results, end of paragraph 1).

 

- **Comment 3:** The study follows by the more precise characterization of the heterogeneity of differentiation response to PB+RA of distinct cell lines, using 3 neuroblastoma models and a prolonged exposure of treatment of 10 days. First, in addition to their distinct genetic background, could the authors precise the different origin of the three selected NB models ? Primary tumor or metastases; before or after treatment; grade; primary tumor location (adrenal gland or sympathetic ganglia) etc… As neuroblastoma is a disease well known for its heterogeneity and so possibly “stalled at different stages of developmental differentiation” as rightly mentioned by the authors in the introduction, could this point be discussed because possibly explaining the obtained results? Minor point: “These cell lines represent a mixture of genetic background” should be replace by “These cell lines represent different genetic backgrounds”.
- **Author response:** Thank you for this interesting point. We have corrected the wording to ‘represent different genetic backgrounds.’ We have also now included available cell line details in the text with references (Results, paragraph 2). We have also included more detail in the discussion as to how the different features of the cell lines may influence their differentiation responses (Discussion, end of paragraph 2).

 

- **Comment 4:** To illustrate distinct differentiation states after treatment, they looked at the cellular morphology (Figure 2A). Whereas the IMR-32 cell line seems only differentiating towards a neuronal fate, BE2C and 5Y showed other phenotype that is not easy to recognize using phase-contrast images. Could the authors find a way to quantify the changes of morphology ? IF maybe ? Photos of control cells at confluence do not allow to really evaluate changes of morphology and may be replace.
- **Author response:** We agree with the Reviewer that further exploration of the cell types emerging from PB+RA treatment of each cell line could be performed. As detailed in the above comment it would be ideal if we could perform a full characterisation of the emerging cell types using single cell techniques such as single cell RNA-seq, however, this is unfortunately not possible. This would allow an accurate identification and comprehensive characterization of the cells emerging following PB+RA treatment, using the markers identified for validation by ICC and flow cytometry. We have expanded on these future work suggestions in the discussion and invite this for further research (Discussion, paragraph 5). Regarding the differentiation images, additional low-confluence images were not provided as these experiments are a follow-on from previous studies where live imaging is performed to track the cells from day 0 of treatment [7]. These movies show clearly the morphology of the cells prior to treatment, which we have clarified in the text for the readers’ reference (Figure 2 legend).

 

- **Comment 5a:** Additionally, they looked at the expression of selected genes by RT-qPCR to evaluate the potential of differentiation in distinct normal cell types of the sympathetic nervous system of the tumor cell lines treated with PB+RA. How these genes have been selected ? It is quite difficult to be certain of a way of differentiation based on the expression of one marker, and at the bulk level. Of note, adrenal cortical cells are coming from a complete different tissue of origin than the adrenal medulla or the sympathetic ganglia, so could the authors justify the use of CYP11A1 ?
- **Author response:** The differentiation markers shown in Figure 2 were selected using marker genes of adrenal medullary cell populations referenced in Jansky et al [1].  NEFM, GAP43 and SYT5 were chosen as markers of sympathetic neuroblasts. PNMT, the enzyme catalyzing methylation of norepinephrine to form epinephrine, was chosen as a marker of mature chromaffin cells, ACTA2 as a marker of myofibroblasts, MYH3 as a marker of myocytes, and CYP11A1 as a marker of adrenal cortical cells. This has now been clarified in the text (Results section, “Differentiation patterns of adrenergic neuroblastoma cell lines correspond to changes in ECM substrate expression“). We note that CYP11A1, as well as MYH3 and ACTA2, mark populations outside of the sympathoadrenal lineage - this is indeed interesting and could suggest malignant transformation at an early stage of neural crest development from bipotent or mesenchymal lineage precursors, and/or plasticity of neuroblastoma cells (26) - we have discussed this further in the text (Discussion, end of paragraph 2). We agree that it would be excellent if we could have performed a full characterisation of the emerging cell types, encompassing all cell type markers and transcription factors in Jansky et al [1] and more, using single cell RNA-seq - we have expanded on these future work suggestions in the discussion and invite this for further research (Discussion, paragraph 5).

 

- **Comment 5b: **How many times this experiment has been conducted ? Does “3 biological replicates” mean 3 independent experiments with how many technical replicates ? To better compare responses between cell lines and to conclude about inter-cell line heterogeneity, it would be necessary to compare also their basal level of expression.
- **Author response:** Three biological replicates were performed for each cell line, corresponding to three independent passages of each cell line. As stated in the ‘Statistical Analysis’ section of the Methods, ‘Biological replicates were considered as different passage numbers of the same cell line plated in independent experiments’. This has been further clarified in the text of the figure legends for Figures 2 and 3. Regarding the comparison of basal levels, the experiments were performed in a manner to compare DMSO with PB+RA treatment within each cell line. We therefore make conclusions as to whether fold changes in gene expression are seen within each line in response to PB+RA treatment. Comparison of the basal gene expression levels would indeed be also interesting, however is beyond the scope of the manuscript.

 

- **Comment 6:** Whereas the IMR32 and the 5Y seem mainly able to go towards a neuronal differentiation, the BE2C seems able to express genes of several cell types from adrenergic to mesenchymal phenotypes (ACTA2, ITGA5, FN1, COLs etc…). Interestingly this cell line has been previously used to study NB tumor cell plasticity, cells being able to shift from a noradrenergic towards a mesenchymal identity. Why the authors did not address the summary of literature on this subject or at least discuss this point? When cells after treatment change phenotypes, in what degree can we distinguish differentiation versus plasticity ?
- **Author response:** This is an interesting point and we thank the Reviewer for prompting us to look further. We have now included reference to cell line plasticity (Introduction, paragraph 2) and features of the cell line details (Results, paragraph 2), including their phenotypic characterisations, noting SK-N-BE(2)C as an ‘I type’ cell line. We have also included more detail in the discussion as to how the different features of the cell lines may influence their differentiation responses (Discussion, paragraph 2). In the Discussion we now “speculate that intra-tumoral differentiation heterogeneity could be associated with heterogeneity within the starting tumour; it would therefore be interesting to investigate the differentiation capacity of a larger cohort of cell lines with I, N and S-type phenotypic characteristics” (end of paragraph 2).

  Thank you for the discussion regarding plasticity vs differentiation. In all three cell lines tested, we observe a dramatic decrease in proliferation with PB+RA treatment (seen in Figures 5, S4, S6, 7 and videos of [7]), further evident after 10 days. This is in contrast to the highly proliferative nature of MES-type neuroblastoma cell lines, such as SH-EP, which may be expected if cells were simply interconverting between malignant cell states. It would indeed be interesting to determine if the resulting cell states seen after treatment are stably differentiated, and single-cell RNA-seq to determine markers of the emerging cell types would be extremely useful to resolve this question. Such an experiment is difficult to truly determine in vitro however; we therefore suggest in vivo experiments would be required to assess the terminal differentiation of the resulting cell states, which is beyond the scope of this manuscript. We have now noted the literature on cell line plasticity in the introduction and discuss in the Discussion (paragraph 2) that phenotypic heterogeneity in cell lines may influence differentiation capacity. Furthermore, we have added to the discussion (last paragraph) “Single cell tracking studies would be insightful to determine if heterogeneous differentiation occurs in response to treatment-induced plasticity, or is predetermined by starting cell identity. Finally, it would also be of interest to treat cells for further extended time periods, in vitro and in vivo, as well as investigate whether continued treatment is required for stable differentiation”.  

- **Comment 7:** Finally, authors looked at ECM factors expression, which is inherently linked to cell identity (especially for mesenchymal cells). Again, the pertinence of looking to these particular genes is missing and would help to interpret the data. What is the biological difference between COL3A1, COL5A1 and COL4A2? Is it known that each of this gene is associated to a specific cell type? Same question about LAMB3 and EGFLAM, why the authors selected those genes?
- **Author response:** As discussed in the Results (section “Differentiation patterns of adrenergic neuroblastoma cell lines correspond to changes in ECM substrate expression“) our investigations into ECM factor expression were led by Tsokos et al’s finding [8] that “specific patterns of ECM expression emerged after differentiation, proportional to the normal tissue cell types observed”, particularly laminin, fibronectin and collagen. In addition, “RNA-seq analyses were performed on SK-N-BE(2)C cells treated with DMSO vehicle control, PB, RA or PB+RA for five days [7]. This identified a subset of genes (cluster 4) upregulated in cells treated with a combination of PB+RA, but not PB or RA alone (Figure 1A and C). Gene ontology analyses revealed that these genes were associated with neuronal cell body and extracellular matrix (ECM) cellular components”. Our choice of ECM markers was guided by Tsokos et al and these genes found to be differentially upregulated in PB+RA treated cells. We have now clarified this in the Results text (paragraph 4-5). As mentioned ‘Further exploration could include characterisation of the emerging cell types using single-cell RNA-seq, immunocytochemistry and flow cytometry analyses.’ As well as the emerging cell states, this would enable a full characterisation of ECM factors and their relation to individual cell types. Unfortunately, this wide-scale characterization was not possible due to time and funding constraints; we have added discussion about this as a future area of research (Discussion, paragraph 5).

 

- **Comment 8**: Concerning the conclusion, these results illustrate inter- neuroblastoma cell line heterogeneity of responses to this therapy, in line with the fact that heterogeneity is a well-known characteristic of neuroblastoma. However, pointing towards “ the role of the ECM in these cell fate decisions” is over-interpreted, not shown nor suggested here, as I would rather say that ECM factors here reflect tumor cell identity and not the other way round.
- **Author response**: We thank the reviewer for this thoughtful comment. As in Tsokos et al [8], our data support that differentiation responses correspond to changes in patterns of ECM substrate expression. We agree with the Reviewer that whether changes in ECM substrate expression are a cause or a consequence of differentiation remains to be determined.

As noted, there are indications in the literature that the ECM may direct cell fate choices, and for example reference 36 where increasing ECM stiffness has been found to modulate neuronal differentiation of neuroblastoma cells and reference 37 where ECM topography has been shown to drive a transition between adrenergic and mesenchymal cell types. As noted, ‘The role of the ECM during neural crest development is well-described, with cells interacting with a dynamic ECM in a spatiotemporal manner to direct their correct differentiation and migration. As such, the ECM composition is a key component of many protocols for neuronal differentiation from stem cell states [29]’. In referencing this literature in the first manuscript version, we intended to discuss whether ECM changes are a cause or consequence of cell fate decisions as an interesting topic for further exploration. For example, ‘it would be interesting to further explore whether ECM modification is able to alter neuroblastoma cell identity in response to PB+RA, and if so, whether drugs altering mechano-transduction pathways may offer additional differentiation benefits.’ We have added to the text to clarify this (Discussion, Paragraph 4), acknowledging that our data are an initial observation to prompt further investigation. It would be important to test this in an in vivo system where cumulative effects of cell types will exist, and have further discussed this in the discussion in paragraph 4. We have also modified the text from ‘build a case… for investigating the effect of ECM on neuroblastoma cell fate decisions’ to ‘investigating ECM changes in neuroblastoma differentiation’ (e.g. end of Introduction and end of Results) to reflect this increased discussion.   We thank the reviewer for their helpful comments which have guided submission of version 2 of this manuscript. While we note that the Reviewer quite rightly states that we could take this work further, we are unable to do so because of staffing constraints. However, we feel strongly that it is important to disseminate our findings to allow others to build on the work that we have done and feel that Wellcome Open Research would be an appropriate venue for this. We hope that the reviewer can consider this when evaluating our responses.

# Reviewer report: Reviewer response for version 1

**DOI:** 10.21956/wellcomeopenres.25621.r111423

**Published:** 2024-12-09

**Authors:** Subrata Sinha

**Affiliations:** 1 Biochemistry, All India Institute of Medical Sciences, New Delhi, Delhi, 110029, India

This paper follows from an earlier publication by the group showing that the combination of retinoic acid (RA - that has been extensively used to differentiate neuroblastoma cells), and palbocicblib (PB), an inhibitor of CDK4/6, has an enhanced effect on the neuronal differentiation and the reduction of proliferation of neuroblastoma derived cell lines (Ferguson K et al 2023). This earlier paper had demonstrated differences in the effects of PB on Adrenergic (ADRN) type neuroblastoma cell lines SK-N-BE(2)C, IMR32 and SH-SY5Y (which had features of neuronal differentiation along with reduction of proliferation) and mesenchymal (MES) type cell lines SH-EP and GIMEN, where there was reduction of cell proliferation but no features of differentiation . There were differences across cell lines when increased differentiation by PB plus RA was compared to PB. Another recent publication (Ferguson K et al,  Wellcome Open Research 2024, 9:667) show in an ADRN type cell line, that other drugs of the CDK4/6 inhibitor type have similar effects as PB.

 In this paper, the authors have studied the heterogeneity in the differentiation patterns of ADRN type cells (SK-N-BE(2)C, IMR32 and SH-SY5Y- with differing genetic backgrounds), which had apparently similar features in the responses to PB and RA as shown by Ferguson K et al 2023 .This  heterogeneity of the response is evident from the post differentiation morphology. Based on RNA seq analysis of SK-N-BE(2)C cells exposed to RA plus PB, they are able to show that these cells have the potential to develop into a variety of adrenal medulla lineages. The 3 cell lines had different morphologies initially, and also differed in their levels of gene expression (as measured by qRT-PCR) for different differentiation states on PB and RA exposure.

 One of the main claims of the paper is that the differentiation patterns of ADRN cell lines correspond to changes in ECM substrate expression. They have identified a group of genes in the RNA seq analysis of SK-N-BE(2)C, that were upregulated in response to PB plus RA but not to any one individually. These were initially identified as one of a group of genes related to neuronal cell body and ECM. Of these, selected ECM genes have been studied. To substantiate this claim, in Fig 3 they have presented qRT-PCR of these 3 cell lines exposed to PB plus RA, and almost all the genes depicted, show a differing response. An issue that is not clear is the whether if ECM substrates produced by the cells determine the nature of differentiation, or whether ECM gene expression patterns are associated with the differentiation pattern as an intrinsic cellular property. *In vivo*, ECM is likely to be generated by a variety of cells and it is very likely there is some kind of cumulative effect of the cell type.

 However, the question is, what relevant biological meaning can be drawn from these mRNA expression values. Heterogeneity of gene expression response is very well known, indeed it can occur after multiple passages of the same cell line, let alone in different cell lines, derived from different primary tumours, having differing genetic backgrounds. It is difficult to claim that the differentiation patterns correspond to the ECM gene expression based on this one experiment of qRT PCR values. How can one conclude that these are not random changes in gene expression by the different cell lines? How were these genes selected for performing qRT PCR, and would the results have been different if others were chosen? Also, how many biological replicates for differentiation were performed and what was the consistency of gene expression in each cell line over different sets of differentiation? Again, it is evident from morphology, that the proportion of flat and neurite bearing cells were different post differentiation in the cell lines. So the RT PCR results represent a mixture of post differentiation cell types. At least some indication of the different cell types post differentiation, could be given from morphological counts and staining by IHC or ICC.

 Again the SH-SY5Y response to differentiation seems to be the expression of more MMP 1. What about the other MMPs? Conventional teaching says that high expression of MMPs is associated with invasive malignancies and not differentiation, and publications for this are also available for MMP1. Some references are below:

**MMP1** Overexpression Promotes Cancer Progression and Associates with Poor Outcome in Head and Neck Carcinoma. Zhang W, Huang X, Huang R, Zhu H, Ye P, Lin X, Zhang S, Wu M, Jiang F. Comput Math Methods Med. 2022 Sep 5;2022:3058342. doi: 10.1155/2022/3058342. eCollection 2022.PMID: 36105241 

Downregulation of **MMP1** **functions** in preventing perineural invasion of pancreatic cancer through blocking the NT-3/TrkC signaling pathway. Xu X, Lu X, Chen L, Peng K, Ji F.J Clin Lab Anal. 2022 Nov;36(11):e24719. doi: 10.1002/jcla.24719. Epub 2022 Sep 30.PMID: 36181286 

 This paper is an extension of their previous work and documents the well knows feature of heterogeneity between cell lines. This could have implications for the heterogeneity of response to therapy in human tumors, particularly in the context of differentiation therapy for neuroblastoma.

 The paper is recommended for indexing with a moderate priority. The concerns raised could be addressed experimentally or in the discussions.

Is the work clearly and accurately presented and does it cite the current literature?

Yes

If applicable, is the statistical analysis and its interpretation appropriate?

Partly

Are all the source data underlying the results available to ensure full reproducibility?

Yes

Is the study design appropriate and is the work technically sound?

Partly

Are the conclusions drawn adequately supported by the results?

Partly

Are sufficient details of methods and analysis provided to allow replication by others?

Yes

Reviewer Expertise:

Cancer biology, molecular oncology

I confirm that I have read this submission and believe that I have an appropriate level of expertise to confirm that it is of an acceptable scientific standard, however I have significant reservations, as outlined above.

# Response

**Published:** 2025-08-24

**Authors:** Anna Philpott

**Affiliations:** Cambridge Stem Cell Institute, Cambridge, England, UK

We thank the reviewer for their helpful comments and respond to each of these below.

- **Comment 1:** One of the main claims of the paper is that the differentiation patterns of ADRN cell lines correspond to changes in ECM substrate expression. They have identified a group of genes in the RNA seq analysis of SK-N-BE(2)C, that were upregulated in response to PB plus RA but not to any one individually. These were initially identified as one of a group of genes related to neuronal cell body and ECM. Of these, selected ECM genes have been studied. To substantiate this claim, in Fig 3 they have presented qRT-PCR of these 3 cell lines exposed to PB plus RA, and almost all the genes depicted, show a differing response. An issue that is not clear is whether ECM substrates produced by the cells determine the nature of differentiation, or whether ECM gene expression patterns are associated with the differentiation pattern as an intrinsic cellular property.  In vivo, ECM is likely to be generated by a variety of cells and it is very likely there is some kind of cumulative effect of the cell type.
- **Author response:** We thank the Reviewer for this insightful comment. As in Tsokos et al [8], our data support that differentiation responses correspond to changes in patterns of ECM substrate expression. We agree with the Reviewer that whether changes in ECM substrate expression are a cause or a consequence of differentiation remains to be determined.

As noted, there are indications in the literature that the ECM may direct cell fate choices, and for example reference 36 where increasing ECM stiffness has been found to modulate neuronal differentiation of neuroblastoma cells and reference 37 where ECM topography has been shown to drive a transition between adrenergic and mesenchymal cell types. As noted, ‘The role of the ECM during neural crest development is well-described, with cells interacting with a dynamic ECM in a spatiotemporal manner to direct their correct differentiation and migration. As such, the ECM composition is a key component of many protocols for neuronal differentiation from stem cell states [29]’. In referencing this literature in the first manuscript version, we intended to discuss whether ECM changes are a cause or consequence of cell fate decisions as an interesting topic for further exploration. For example, ‘it would be interesting to further explore whether ECM modification is able to alter neuroblastoma cell identity in response to PB+RA, and if so, whether drugs altering mechano-transduction pathways may offer additional differentiation benefits.’ We have added to the text to clarify this (Discussion, Paragraph 4), acknowledging that our data are an initial observation to prompt further investigation. We agree with the reviewer that it would be important to test this in an in vivo system where cumulative effects of cell types will exist, and have further discussed this in the discussion in paragraph 4. We have also modified the text from ‘build a case… for investigating the effect of ECM on neuroblastoma cell fate decisions’ to ‘investigating ECM changes in neuroblastoma differentiation’ (e.g. end of Introduction and end of Results) to reflect this increased discussion.  

- **Comment 2:** However, the question is, what relevant biological meaning can be drawn from these mRNA expression values. Heterogeneity of gene expression response is very well known, indeed it can occur after multiple passages of the same cell line, let alone in different cell lines, derived from different primary tumours, having differing genetic backgrounds. It is difficult to claim that the differentiation patterns correspond to the ECM gene expression based on this one experiment of qRT PCR values. How can one conclude that these are not random changes in gene expression by the different cell lines? How were these genes selected for performing qRT PCR, and would the results have been different if others were chosen? Also, how many biological replicates for differentiation were performed and what was the consistency of gene expression in each cell line over different sets of differentiation? It is difficult to claim that the differentiation patterns correspond to the ECM gene expression based on this one experiment of qRT PCR values. How can one conclude that these are not random changes in gene expression by the different cell lines?
- **Author response:** Three biological replicates were performed for each cell line, corresponding to three independent passages of each cell line. As stated in the ‘Statistical Analysis’ section of the Methods, ‘Biological replicates were considered as different passage numbers of the same cell line plated in independent experiments.’ We are therefore confident that the changes in gene expression seen are indeed not random events, as they were consistently seen across three independent experiments in different cell line passages. We have now clarified in the text of the figure legends for Figures 2 and 3.

 

- **Comment 3**: Again, it is evident from morphology, that the proportion of flat and neurite bearing cells were different post differentiation in the cell lines. So the RT PCR results represent a mixture of post differentiation cell types. At least some indication of the different cell types post differentiation, could be given from morphological counts and staining by IHC or ICC.
- **Author response:** We agree with the reviewer that further exploration of the cell types emerging from PB+RA treatment of each cell line could be performed. As stated in the discussion ‘Further exploration could include characterisation of the emerging cell types using single-cell RNA-seq, immunocytochemistry and flow cytometry analyses.’ It would be a significant piece of new work to perform a full characterisation of the emerging cell types using single-cell techniques such as single-cell RNA-seq that we would like to have undertaken. This would allow an accurate identification and comprehensive characterization of the cells emerging following PB+RA treatment. However, this is unfortunately not possible due to funding constraints. We have expanded on these future work suggestions in the discussion and invite this as an important area for further research (Discussion, Paragraph 5).

 

- **Comment 4:** Again the SH-SY5Y response to differentiation seems to be the expression of more MMP 1. What about the other MMPs? Conventional teaching says that high expression of MMPs is associated with invasive malignancies and not differentiation, and publications for this are also available for MMP1. Some references are below: MMP1 Overexpression Promotes Cancer Progression and Associates with Poor Outcome in Head and Neck Carcinoma. Zhang W, Huang X, Huang R, Zhu H, Ye P, Lin X, Zhang S, Wu M, Jiang F. Comput Math Methods Med. 2022 Sep 5;2022:3058342. doi: 10.1155/2022/3058342. eCollection 2022.PMID: 36105241, Downregulation of MMP1 functions in preventing perineural invasion of pancreatic cancer through blocking the NT-3/TrkC signaling pathway. Xu X, Lu X, Chen L, Peng K, Ji F.J Clin Lab Anal. 2022 Nov;36(11):e24719. doi: 10.1002/jcla.24719. Epub 2022 Sep 30.PMID: 36181286
- **Author response:** Thank you for this comment and pointing out references we had missed. We have expanded on the interesting observation of increased MMP1 expression in PB+RA treated SH-SY5Y cells in the discussion to include these references (end of paragraph 3). We agree that this is a surprising observation, and surmised that upregulation of a matrix metalloproteinase responsible for degradation of ECM components may link to unique cell fates in this cell line as increasing ECM stiffness has been found to modulate neuronal differentiation of neuroblastoma cells. Further characterisation of the other MMPs would be interesting as part of a more extensive analysis of neuroblastoma differentiation heterogeneity, as detailed in the above comment, but it is not possible for us to undertake this work at this time.

  We thank the reviewer for their helpful comments which have guided submission of version 2 of this manuscript. While we note that the Reviewer quite rightly states that we could take this work further, we are unable to do so because of staffing/funding constraints. However, we feel strongly that it is important to disseminate our findings to allow others to build on the work that we have done and feel that Wellcome Open Research is an appropriate venue for this. We hope that the reviewer can consider this when evaluating our responses.
